# Open-DPSM: An open-source toolkit for modeling pupil size changes to dynamic visual inputs

**DOI:** 10.3758/s13428-023-02292-1

**Published:** 2023-12-11

**Authors:** Yuqing Cai, Christoph Strauch, Stefan Van der Stigchel, Marnix Naber

**Affiliations:** Experimental Psychology, Helmholtz Institute, Faculty of Social Sciences, Utrecht University, Heidelberglaan 1, 3584 CS Utrecht, The Netherlands

**Keywords:** Pupillometry, Modeling pupil size, Pupillary light response, Open-DPSM

## Abstract

Pupil size change is a widely adopted, sensitive indicator for sensory and cognitive processes. However, the interpretation of these changes is complicated by the influence of multiple low-level effects, such as brightness or contrast changes, posing challenges to applying pupillometry outside of extremely controlled settings. Building on and extending previous models, we here introduce Open Dynamic Pupil Size Modeling (Open-DPSM), an open-source toolkit to model pupil size changes to dynamically changing visual inputs using a convolution approach. Open-DPSM incorporates three key steps: (1) Modeling pupillary responses to both luminance and contrast changes; (2) Weighing of the distinct contributions of visual events across the visual field on pupil size change; and (3) Incorporating gaze-contingent visual event extraction and modeling. These steps improve the prediction of pupil size changes beyond the here-evaluated benchmarks. Open-DPSM provides Python functions, as well as a graphical user interface (GUI), enabling the extension of its applications to versatile scenarios and adaptations to individualized needs. By obtaining a predicted pupil trace using video and eye-tracking data, users can mitigate the effects of low-level features by subtracting the predicted trace or assess the efficacy of the low-level feature manipulations a priori by comparing estimated traces across conditions.

**Supplementary Information:**

The online version contains supplementary material available at 10.3758/s13428-023-02292-1.

## Introduction

Pupillary responses are sensitive and versatile indicators of physiological changes accompanying or underlying human cognition (see Binda & Gamlin, 2017; Mathôt, 2018; Strauch et al., 2022a; Wilhelm, 2008 for reviews). When measuring pupil size in daily life, a complex pattern of constrictions and dilations emerges. What makes this signal so complex? Besides steady-state factors affecting baseline pupil size, such as age and overall luminance (Watson & Yellott, 2012), pupil size also responds to all kinds of sensory and cognitive events (Strauch et al., 2022a). This multitude of parallel factors affecting pupil size makes it challenging to dissociate the different components. For instance, pupillary light responses assessed by a neurologist (Wilhelm, 2008) might be confounded by attention or arousal modulations. Psychologists or cognitive neuroscientists, in turn, are usually interested in such attentional effects exclusively, and thus aim to eliminate possible confounds due to light levels. To study one isolated aspect, such as cognitive modulations, highly controlled stimulus materials and laboratory settings are usually adopted. Here, we introduce “*Open dynamic pupil size modeling*” (Open-DPSM), a convolutional model capable of modeling the effects of visual low-level features on pupil size changes using dynamic and complex stimuli such as videos with unconstrained eye movement, which in turn allows estimation of attentional effects. We make Open-DPSM fully available with open-source Python scripts and an accessible graphical user interface to the community.

### Modeling pupil size

As pupil size changes reflect an intertwined combination of low-level visual events and higher cognitive events, it is necessary to mitigate the influence of unwanted confounders. Brightness changes, eliciting the pupil light response (PLR), are the most prominent low-level features. Besides stringently controlling luminance, modeling is used to estimate the PLR to complex and dynamic stimuli from luminance changes. The subtraction of this modeled trace from measured pupil size then yields a more “purified” trace of cognitively driven pupil size changes (David-John et al., 2018; Fanourakis & Chanel, 2022; Napieralski & Rynkiewicz, 2019; Raiturkar et al., 2016; Wong et al., 2020). Such models can be roughly divided into two categories: steady-state models to estimate PLR under equilibrium state (Moon & Spencer, 1944; Raiturkar et al., 2016; Watson & Yellott, 2012) and dynamic models incorporating the PLR under transient influence of the light (Fan & Yao, 2011; Longtin & Milton, 1989; Pamplona et al., 2009; Usui & Hirata, 1995). As expected, for dynamic and constantly changing stimuli, dynamic models are found to be more effective for the accurate estimation of pupil size change, even though they aren't widely adopted yet (Fanourakis & Chanel, 2022). The adaptation of many of these models is challenging, as they incorporate the biomechanical functions of muscles and the feedback loop from the brain that are thought to control pupillary responses, which require complicated computations or additional experimental conditions/calibrations to estimate free parameters and individual differences (Soleymani et al., 2012; Zandi & Khanh, 2021).

**Fig. 1:**
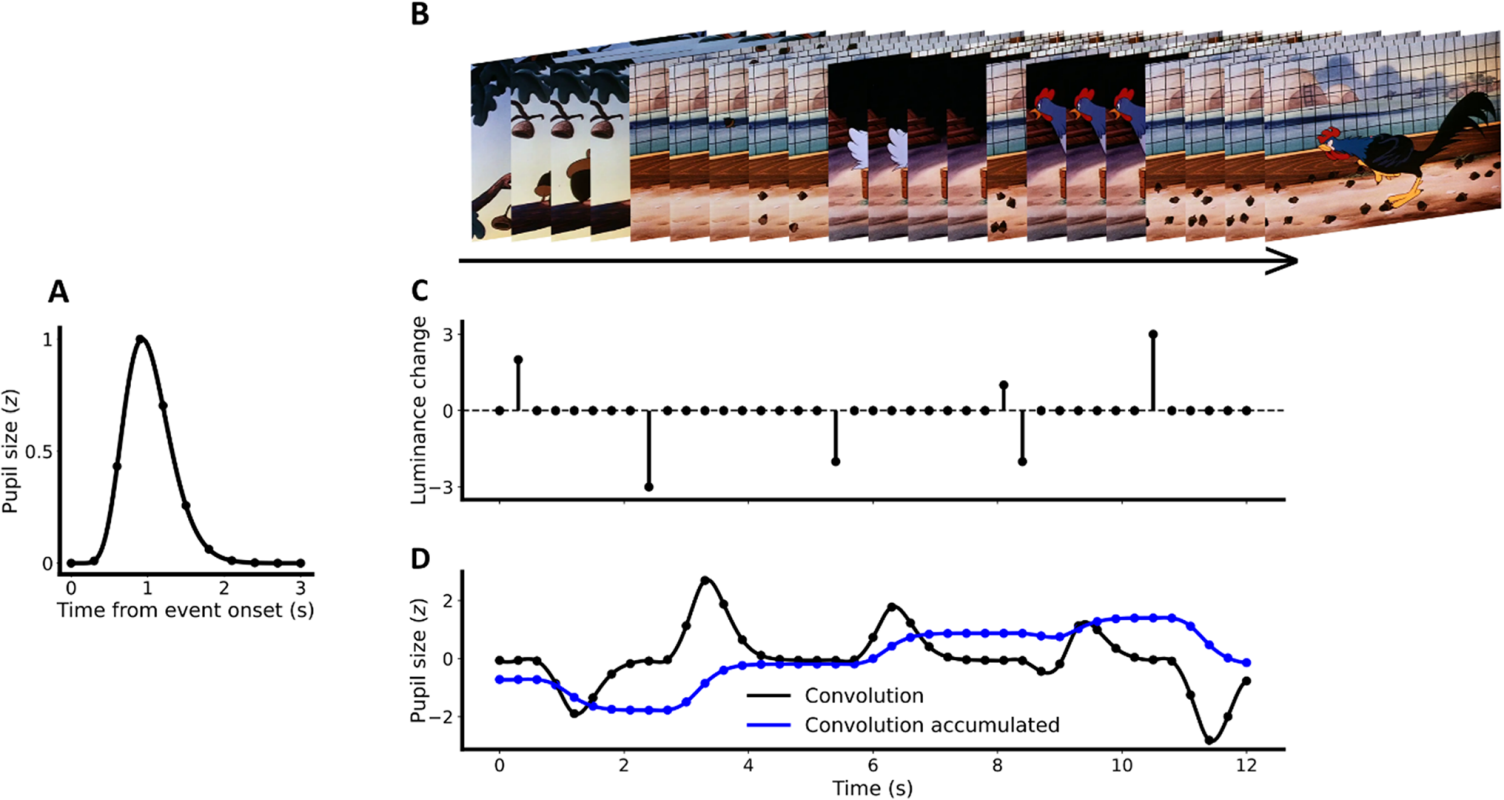
Convolution of response function and visual events. **A** Pupil response function (RF) for convolution, based on Hoeks and Levelt (1993). **B** Examples of several movie frames. **C** Exemplary data of luminance changes over time, which can be either positive (change to brighter) or negative (change to darker). Changes are extracted as difference of luminance between two consecutive images in B and then aggregated to time-series data. **D** Results of response function in A convoluted with the time-series luminance changes in C, resulting in transient (temporary) changes (*black line*) and accumulated (sustained) changes (*blue line*). Note that positive values in C indicate positive luminance change (brighter), the predicted pupil size in D therefore changes to the opposite direction of A

Alternatively to such biophysiological models, studies have adopted convolutional approaches to model pupil dynamics to both higher-level cognitive events and lower-level visual events. As pupil size exhibits a relatively slow response to the perceptual and cognitive events that drive it, pupil size at any given time reflects the consequence of multiple, overlapping, distinct perceptual and cognitive events (Denison et al., 2020; Wierda et al., 2012). Assuming that a linear time-invariant system underlies pupillary changes, two consecutive events elicit responses that overlap with each other in time, resulting in an overall pupil response reflecting the sum of both responses. A gamma-shaped response function (RF) (Fig. 1A) is usually adopted to approximate each of those delayed responses to any events (e.g., changes in stimulus material as shown in Fig. 1B, C). More specifically, as schematically outlined in Fig. 1, each event is convolved with a response function. The “stronger” the event (Fig. 1C), the higher the amplitude of the pupillary response (Fig. 1D). Visual events, such as brightness changes, would influence pupil size not only transiently, but also leads to a sustained new size (e.g., a constricted pupil if brightness is increased). To model this sustained response, the pupillary response is further cumulated (Fig. 1D blue line). A number of studies have exploited this convolutional nature to either model the effects of cognitive events on pupil responses or to retrieve the timing and strength of cognitive events using deconvolution techniques (de Gee et al., 2014; Denison et al., 2020; Hoeks & Levelt, 1993; Kang & Wheatley, 2015; Knapen et al., 2016; Wierda et al., 2012). On the other hand, Korn and Bach (2016) also used the convolutional approach to model luminance changes. To this end, two response functions were fitted to (a) model sustained changes to luminance in general and (b) model temporary 'overshoots' to increases in luminance. These overshoots consist of short-lasting pupil constrictions after luminance increments that are more strongly than expected if only taking into account sustained luminance changes. It is well possible that such constrictions could potentially be the result of a low-level feature other than a sustained luminance change. The effect of luminance can be modeled out as a nuisance covariate to allow for the study of cognition-induced pupil size changes (for existing software, see Psycho Physiological Modelling (PsPM); Korn et al., 2017). Other than luminance changes, convolution should, in principle, also allow modeling the effects of other low-level feature changes, such as contrast, color, or spatial frequency (see Strauch et al., 2022a for a review).

### Current model

Although Korn and Bach (2016) successfully captured the characteristics of pupillary responses to *simple* and *temporally separated* luminance changes, it is unknown whether this approach can predict pupil size changes when observers watch more *dynamic, complex,* and *temporally overlapping* visual changes, such as in a movie. Building on existing modeling and theoretical advancements, Open-DPSM directly applies the convolution approach to the extracted visual events of movies over time. We will additionally demonstrate that Open-DPSM succeeds in predicting pupil size to highly dynamic visual input flexibly without fixation restrictions by incorporating: (i) transient response to contrast changes; (ii) weighted contribution of different visual field regions and (iii) gaze-contingent visual events extraction. Why and how well these features improve the modeling results will be incrementally extended on throughout this manuscript.

## Visual event extraction

To model pupil size dynamics, visual events (i.e., luminance changes) are extracted from the stimulus material first (here: movie frames). For each movie frame, a three-color channel (red, green, blue; RGB) image (Fig. 1B) is loaded in an 8-bit matrix. Then, luminance changes are computed as described in the following.

### Convert RGB to CIELAB

To better match how the human visual system “perceives” computer images, each RGB image frame is converted to CIELAB space (International Commission on Illumination, 2004) with the function “cvtColor” in the python package OpenCV. The first channel L* of the CIELAB space is used as the input value to calculate luminance on the screen.

### Gamma correction to convert L* to physical luminance

The L* extracted from the image represents the relative luminance on the scale of black (0 as minimum value) to white (255 as maximum value). Note that these values do not represent the absolute amount of physical light emitted by the monitor, as the monitor scales the input nonlinearly to luminance using a gamma function. Typically, OLED screens apply a gamma value of 2.2, which prevents luminance saturation and thus enhances the aesthetical appearance of the images (Cooper et al., 2013). The higher this gamma value, the stronger the nonlinearity and darkening of lower input values. Physical luminance in candela per square meters (cd/m^2^) is hence computed by applying a gamma correction to L* input. This physical luminance in cd/m^2^ will be used to model pupil size change (which will be referred to as luminance henceforth) because a roughly linear relationship between the physical luminance change in candela per square meters (cd/m^2^) and the amplitude of pupillary response to luminance change was found within the current limited range of luminance changes in movies (see Supplementary Fig. 1). Note, however, that more extreme luminance ranges result in non-linear links due to flooring/ceiling effects (see Watson & Yellott, 2012).

### Luminance change

The change in overall luminance per frame is calculated by taking the mean difference in luminance across all pixel values between two frames. More specifically, for our movies with a 25-Hz frame rate, we calculate the difference in every two movie frames (i.e., two frames separated by 80 ms) to mimic the sampling rate of the human visual system (Intraub, 1981; Potter, 1975; Potter et al., 2002). The changes in luminance at the first two frames (or at times 0 s and 0.04 s) were calculated by subtracting the luminance of a homogenously black image from these initial frames.

## Dataset

### Participants & stimulus material

To train and test our models, eye-tracking data were collected from *n* = 15 participants (*M*_Age_ = 24.43; *SD*_Age_ = 2.43 years; seven males) who watched twenty movie clips of 60 s each in random order. Movie clips were selected from a larger set published earlier (Gestefeld et al., 2020). The subset of movies, including 18 cartoon/animated movies and two movies with real actors, were selected because these contained relatively many luminance changes.

### Apparatus

A large OLED65B8PLA LG 65” TV (145 by 80 cm (88.1° by 56.1° visual angle) displayed the movies at a resolution of 1920 by 1080 pixels and a refresh rate of 100 Hz. The maximum 100% brightness of the TV was 212 cd/m^2^ and the gamma value is 2.2. Participants sat 75 cm away from the screen while they kept their head fixed in a chin- and forehead rest. The built-in speaker of the TV played the audio of the movie at an average loudness of around 50 dB (range 40–70 dB).

Eye movement and pupil size data of both eyes were recorded with a tower-mounted EyeLink 1000 (SR Research) at 500 Hz. The eye tracker was set to circular fitting of the pupil and its diameter served as the pupil size measure. The TV connected to a separate desktop computer with an installed Python-based PsychoPy package that allowed controlled movie presentations and EyeLink communication (version 2022.2.4, Peirce et al., 2019).

During the experiment, the only source of light apart from the stimulus-displaying TV was the EyeLink communication monitor, resulting in less than 1 Lux (lx) at eye position.

### Procedure

The participants watched the movies without further task instructions other than keeping their heads still. An eye-tracker calibration (five-point calibration and validation) was performed before the start of the first trial and every five trials (i.e., movie clips) during the experiment. Participants started trials/movies self-paced. If a participant moved her/his head, a recalibration was performed, and participants watched the movie again.

## Modeling pupil size

### Benchmark models

Before describing the more advanced steps in building Open-DPSM, we introduce two benchmark models for pupil size predictions, adjusted for continuous video input and modeling: (1) a prior-less polynomial model, and (2) an adaptation of the model by Korn and Bach (2016). To optimize the model, the root mean squared error (RMSE) was minimized by fitting parameters using ordinary least square minimization with a Nelder–Mead simplex search algorithm (“minimize” function in Python’s *scipy* package^1^). We used explained variance (*R*^2^) between actual and fitted pupil size change to evaluate model performance.

#### Polynomial model

The polynomial model served as a test on whether a model with the same number of free parameters as the later introduced models can account for the changes in pupil size across time. We fitted a quintic (five-degree) polynomial, corresponding to the number of parameters in the later described ‘Contrast response model’, to all the pupil data traces of all the movies for each participant with one set of best-fitting parameters.

Unsurprisingly, the performance of the polynomial model was poor (*R*^*2*^ < 0.001).

#### Extending Korn and Bach (2016)

**Fig. 2:**
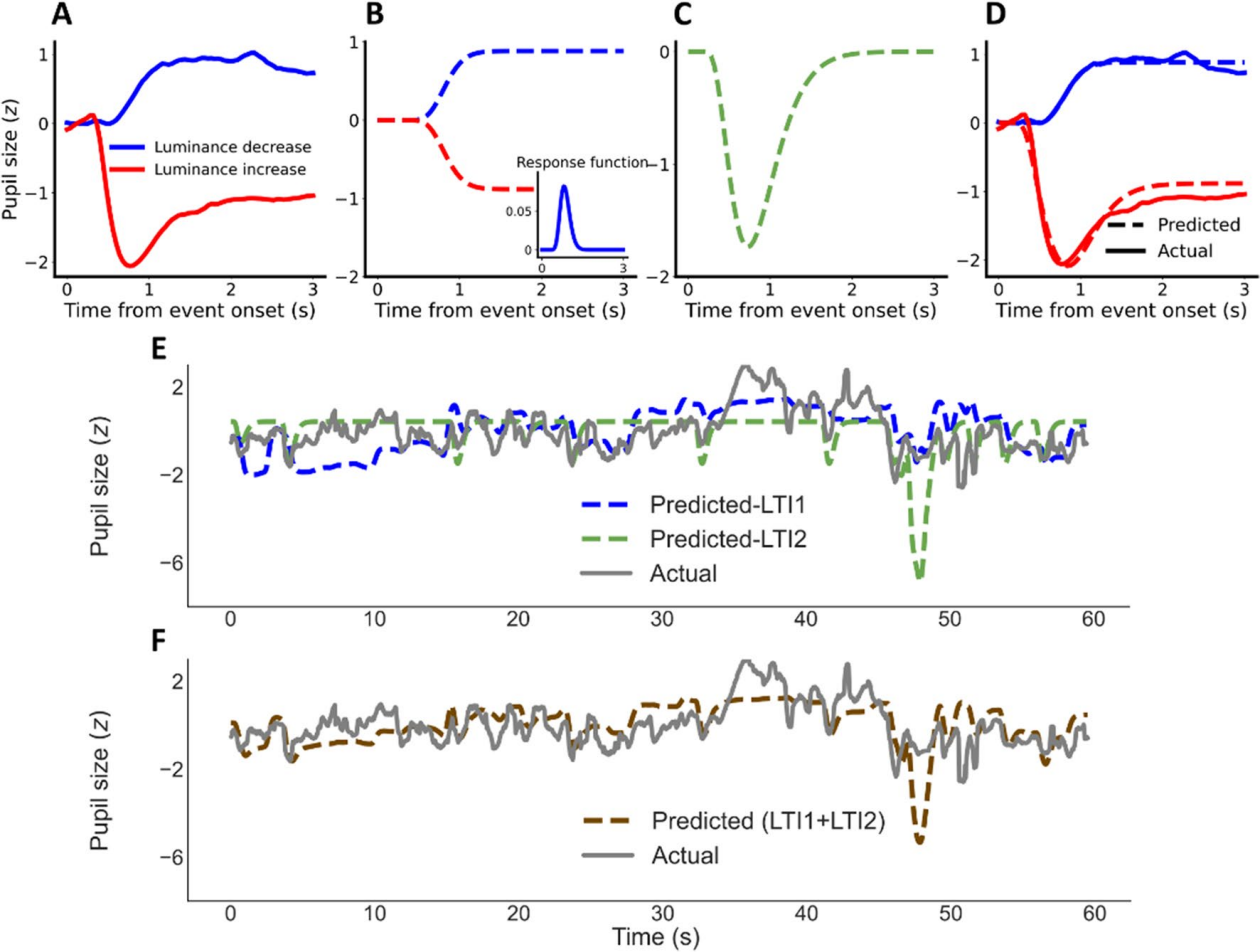
Adapting Korn and Bach (2016) to model event-related pupil responses with RFs and applying them to continuous time series. **A** Grand mean of event-related pupil responses to luminance increases (constriction, *red*) and decreases (dilation, *blue*); **B** Fitted cumulative event-related pupil responses (see subplot for original derivative) for LTI1 dark (*blue*) and bright (*red*) events. Note that Korn and Bach (2016) only used dilation data to model the LTI so the pupil constriction here (*red*) was created by inversing the modeled pupil dilation pattern. **C** Predicted difference between dilation and constriction of the LTI2 to capture the overshoot in response to luminance increases. **D** Actual (*solid*) and predicted (*dashed*) event-related pupil responses for constrictions (*red*) and dilations (*blue*), with the prediction of constriction based on the combination of the two RFs in panel **B** and **C**. **E** Exemplary convolution of the two RFs of both LTI systems to continuous luminance changes (*dashed blue* and *green*), in comparison to actual pupil data (*solid grey*). **F** A weighted combination of the two LTI systems (*dashed brown*) and the comparison to actual pupil data (*solid grey*)

Korn and Bach (2016) suggested that pupil size changes in response to luminance changes can best be explained by two distinct linear-time-invariant (LTI) systems, each employing a unique RF. The RF in the LTI1 modeled the sustained pupillary dilation and constriction to luminance change (Fig. 2B) and the RF in the LTI2 captured the extra ‘overshoot’ in constriction responding to luminance increases (i.e., the residual difference between the red lines in Fig. 2A, B) that was not captured by LTI1. Korn and Bach (2016) tested the model using static and discrete stimuli with alternating luminance levels every 5 s. The two RFs were first fitted to event-related pupil response and then pupil prediction was evaluated with time-series luminance events. To predict pupil size with continuous and overlapping visual events, the model by Korn and Bach (2016) was extended to apply to movie-watching data.

##### Modeling methods and results

*Event-related pupil response extraction.* To extract event-related pupil responses, events of luminance changes in the movies had to be detected first. As we intended to mimic the predefined and rather large discrete luminance changes in Korn and Bach (2016), the luminance changes with varying intensities throughout the movie were determined post hoc with a threshold (all the luminance changes below ±3 cd/m^2^ were removed; final luminance change magnitudes ranging from – 45.8 to 46.3 cd/m^2^). Similar to Korn & Bach’s stimulus design, events in too short succession (below 1 s) were removed, which reduced crosstalk between consecutive events. Event-related pupil responses were extracted by segmenting the pupil time-series data into 3-s segments using each event as the onset. Each event-related pupil responses were baseline corrected by subtracting the average pupil size of the first 250 ms after event onset, as the pupil takes approximately 250 ms to start responding to a luminance change. Event-related pupil responses were further divided by the overall standard deviation across all event-related pupil responses of all participants to be further z-standardized. After extracting all luminance-related pupil responses, the grand mean of dilation (Fig. 2A blue trace) and constriction (Fig. 2A red trace), and the difference between them were calculated.

**Fig. 3:**
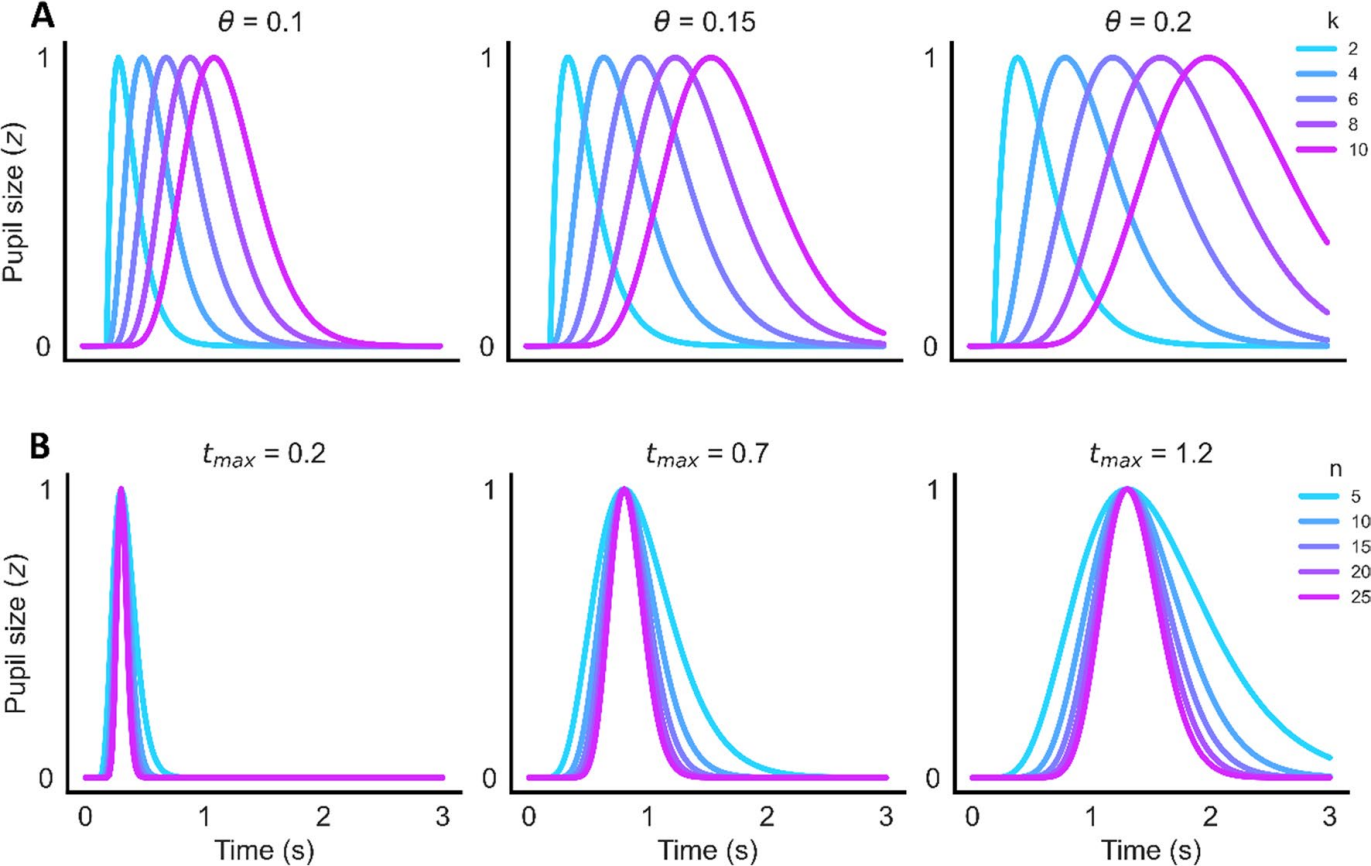
Exemplary pupil response functions. **A** The illustrations of Gamma probability density functions (Korn & Bach, 2016) and **B** Erlang gamma functions (Hoeks & Levelt, 1993) with different combinations of values for the free parameter. For both functions, an unchanged pupil size period of 200 ms is set and all RFs are normalized to a maximum value of 1 for illustration purposes. *Θ* and *k* in Gamma probability density function interactively control for timing and shape of the RF. *t*_*max*_ exclusively controls for timing of the peak in Erlang gamma function, and *t*_*max*_ and *n* for shape/width

*Modeling the pupillary responses with two LTI systems.* Following Korn and Bach (2016), the two RFs were then fitted to the grand mean of dilation and the difference between the dilation and constriction respectively, using the gamma probability density function. This function has three free parameters (see formula (1), *c* standing for the peak amplitude, and *k* and *Θ* for the shape and time of peak amplitude collectively. Also see Fig. 3A for examples of this function across different parameter combinations). The starting point of the response function was set to 200 ms past the event.

1 $$d(t)=c/\left({\theta}^k\varGamma \kern0.5em (k)\right){t}^{k-1}\exp \left(-t/\theta \right)$$

As the LTI1 reflected the sustained response to luminance change, the grand mean of pupil dilation was modeled by the cumulative of the first RF (*c* = 0.04, *k* = 14.1, *Θ* = 0.05; Fig. 3B). The second RF was approximated by another gamma probability density function to model the difference between constriction and dilation (Fig. 2C*; c* = 1.23, *k* = 4.7, *Θ* = 0.14). A weight to the LTI2 was fitted to determine the relative amplitude (*weight* = 1.003) of the second RF as compared with the first RF. The combination of the two RFs explained almost all variance in both the dilation (*R*^*2*^ = 0.97) and constriction (*R*^*2*^ = 0.94) patterns of the event-related pupil responses (Fig. 2D).

*Evaluating pupil prediction with time series data.* The two RFs were convolved with time-series data of all luminance changes and luminance increases per movie, respectively (for an example, see Fig. 2E), and were then combined to a weighted sum (Fig. 2F), which explained the variance in time-series of pupil size above chance, albeit much less so (mean of all participants: *R*^2^ = 0.197, SD = 0.059) than for the event-related responses. Note that the LTI1 contributed much more strongly to explaining variance (*R*^2^ = 0.191, *SD* = 0.0512) than the LTI2 (*R*^2^ = 0.046, *SD* = 0.022).

###### Alternative response function

Besides the gamma probability density function used in Korn and Bach (2016), the Erlang gamma function was previously proposed to fit event-related pupil responses well (Hoeks & Levelt, 1993). This function can be considered more intuitive because the timing of the peak amplitude is determined by a single parameter (formula (2)) rather than being the result of a complex interaction between two parameters as in the gamma probability density function.

2 $$h(t)={t}^n{e}^{- nt/{t}_{max}}$$

The Erlang gamma function relies on two parameters: *t*_*max*_*,* determining the timing of peak amplitude, and *n* the shape/width of the pupil response function (Fig. 3B). A third multiplication parameter is added to this function to control the relative amplitude of the peak. After applying the Erlang gamma function to our data, the model performs comparably to the gamma probability density function (*R*^*2*^ = 0.197, *SD* = 0.058). As the Erlang gamma function is easier to interpret, we only adopted this function in the following models (Results of models using the gamma probability density are reported in Supplementary Table 1 as a complement, both response functions can be called in Open-DPSM).

### Open-Dynamic Pupil Size Modeling (*Open-DPSM*)

We next introduce *Open-DPSM*, a convolutional model for pupil size changes to dynamic visual stimulation. Open-DPSM incorporates three major features discussed in the following sections in more detail: (1) Integration of a transient contrast-based pupil response in addition to a sustained response to luminance change; (2) Separate weights of different image regions in their contribution to pupil size changes; (3) Gaze-contingent feature extraction and modeling. For each step, a brief motivation is outlined, steps are described and model improvements are reported.

#### Integration of a contrast response function

The phenomenological “overshoot” responding to increases in luminance, which results in “undershoots” by modelled sustained luminance responses (Korn & Bach, 2016), can be alternatively interpreted as the orienting response of pupil (Barbur et al., 1992; Gamlin et al., 1998; Hu et al., 2019; Kanari & Kaneko, 2021; Naber et al., 2011; Nakano et al., 2021; Slooter & van Norren, 1980; Young et al., 1995; see Strauch, 2022a for a review). Visual events, including changes in luminance, contrast, color, or any other visual feature evoke a temporary constriction that scales with the salience of the event as shifts of attention (Wang et al., 2014; together with a sometimes small faster dilation preceding the constriction). Such short-lasting constrictions can overlap with sustained responses to luminance changes. As such, we took luminance contrast (absolute value of luminance change) as a measure of visual change independent of the direction of luminance change (see Discussion for the potential impact of features beyond contrast). We hypothesized that both luminance increases and decreases would elicit a separate transient pupil component which would scale with the degree of luminance contrast change. It is important to note that pupil dilations to luminance decreases are also likely to contain such a contrast-dependent component (Barbur et al., 1992). However, its effect may not always be directly visible as the overlapping, sustained dilation may conceal this transient constriction, although the latter may result in an apparently delayed pupil response. When the transient constriction surpasses the sustained dilation, a counterintuitive observation of constriction response to darkness can also emerge, which is consistent with such observations in rapid presentations of flashes with different illuminance levels (see Figure 8 in Korn & Bach, 2016, and Supplementary Fig. 2). We next extend the model based on this idea.

##### Modeling methods and results

**Fig. 4:**
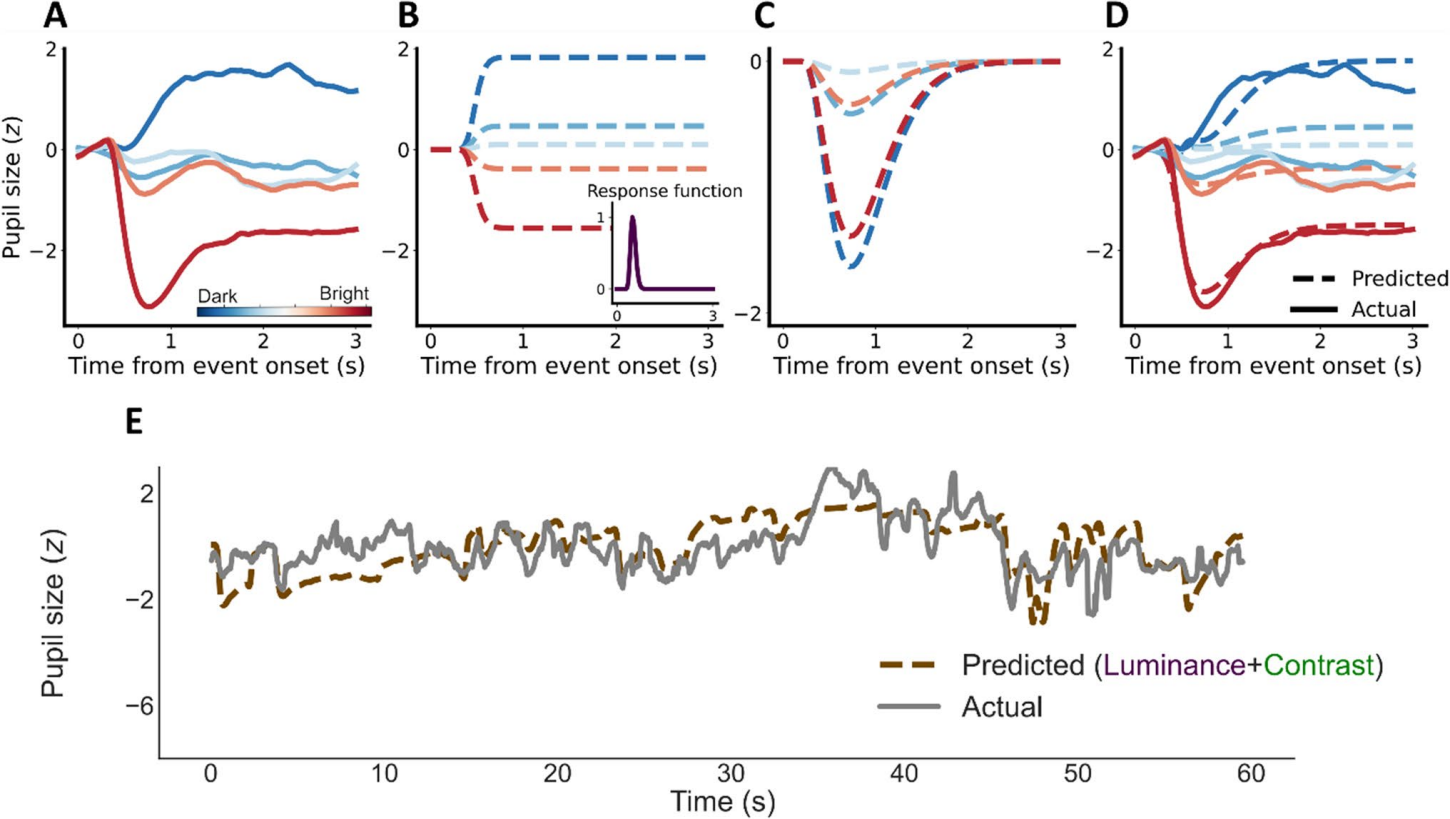
Contrast response model. **A** Event-related pupil responses binned to five levels of luminance changes. **B** Predicted event-related pupil responses with the RF (*subplot*) for sustained responses to luminance changes. **C** Predicted event-related pupil responses for transient responses to contrast changes. **D** Actual (*solid*) and predicted (*dashed*) event-related pupil responses combined the two RFs in B and C with a weight. E Exemplary combined weighted prediction (*dashed brown*) and actual pupil size change (*solid grey*)

This “contrast response model” differed from the modified version of Korn and Bach (2016) described above in two aspects. First, the second transient response function was modeled based on contrast and not used to model the “overshoot” in responses to luminance increases exclusively. Second, the amplitude of this response scaled with the degree of contrast change as more salient events should evoke stronger pupil constrictions. As before, we first calculated event-related pupil responses to different magnitudes of sustained luminance changes. We binned the entire range of possible magnitudes of luminance changes into five separate bins using percentiles and calculated the average event-related pupil response per bin (Fig. 4A; 0–20 much darker; 21–40 slightly darker, 41–60 very small change or no change; 61–80 slightly brighter, 81–100 much brighter; see Supplementary Table 2 for the average and range of luminance change amount per bin).

The five sustained luminance responses were then modeled with the cumulative RF (Fig. 4B). The contrast responses were modeled with a transient RF (Fig. 4C). Similar to the previous models, optimization was used to find the best-fitting parameters (RF for luminance change: *n* = 13.7, *t*_*max*_ = 0.28s; RF for contrast change: *n* = 3.0, *t*_*max*_ = 0.53s; Relative weight of contrast change as compared with luminance change: 0.42; *R*^*2*^ = 0.893, Fig. 4D).

The two RFs were then convolved with luminance and contrast changes respectively and combined into a single weighted prediction (Fig. 4E). The model performance was evaluated the same way with time-series data of all luminance changes, and the result outperformed the last model (*R*^*2*^ = 0.242, *SD* = 0.069; *t*(14) = – 12.901, *p* < 0.001).

##### Convolving RFs directly with luminance and contrast changes

**Fig. 5:**
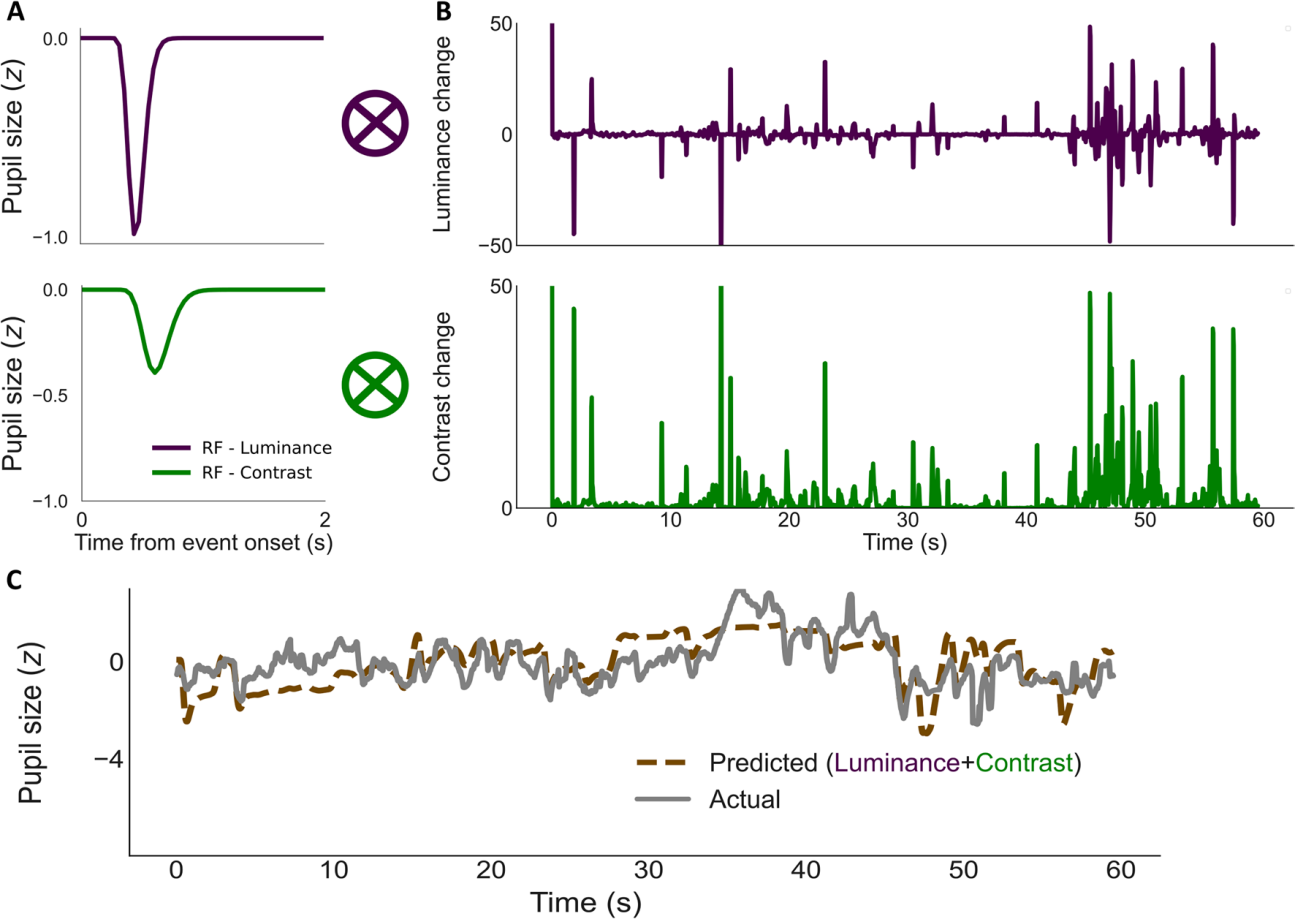
Temporally continuous modeling method**.** **A** Two exemplary RFs to model luminance and contrast change each with two free parameters convolved with **B** the luminance (*purple*) and contrast changes (*green*) over time. Note that the convolutional result for luminance changes was cumulated, as in Fig. 1D (*blue line*). **C** Prediction (*dashed brown*) and actual pupil data (*solid grey*)

The aforementioned models mandated a two-step procedure: (1) Fit two RFs to the event-related pupil responses and (2) convolve the RFs to time series of visual events to predict continuous pupil size changes. Although the contrast response model described above already outperformed the polynomial model and Korn and Bach (2016), several constraints remained: Firstly, the detection of events required arbitrary luminance thresholds and an arbitrary number of bins that segregates the range of luminance levels. Secondly, this method cannot account for covariations and interactions between effects of parallel visual events (i.e., luminance and contrast). This would also impede later model extensions for other types of visual events (see Discussion; Gamlin et al., 1998; Kimura & Young, 1995; Oster et al., 2022; Portengenet al., 2023a; Young et al., 1993). Subsequently, we modeled continuous pupil size data using the raw rather than threshold-dependent time-series data of luminance and contrast changes and fitted the RFs directly to time-series data instead of fitting RFs as a priori on event-related pupil responses (Fig. 5A, B). This direct convolution of visual events with RFs would also allow for more flexibility in other manipulations, such as assigning weights to denote relative contributions of visual field regions (see next model). We hereby refer to this modeling procedure as “temporally continuous” and the previous step-wise modeling as “temporally discrete”.

The two separate response functions were directly convolved with each trace of luminance and contrast changes, respectively, and parameters were optimized as before. Except for direct convolution of time-series data, this method was different from the previous fitting procedure in two ways: Firstly, since no thresholding or cutoff was required, a larger amount of data was available, and hence, RFs were fitted per participant and results across participants were able to be compared (see Supplementary Fig. 3A, B for selected parameters in RFs across all participants). Secondly, as the parameters of the RFs were determined by fitting the time-series data directly and the fitted result cannot be visually examined, we used a repeated cross-validation procedure to avoid overfitting and ensure the robustness of the model. Twenty trials of each participant were divided randomly into training (70%) and testing sets (30%) and this procedure was repeated over five iterations. All following models would maintain those adaptations and the average results of only the testing sets across the five folds would be reported.

Model performance again improved significantly (*R*^*2*^ = 0.261, *SD* = 0.069; *t*(14) = 3.432, *p* = 0.004).

#### Regionally weighted

The influence of visual events on pupil size changes depends on the location of their appearance in the visual field, a phenomenon termed a (pupillary) visual field anisotropy (e.g., Ferree et al., 1933; Kardon et al., 1991; Naber & Nakayama, 2013; Portengen et al., 2021). More pronounced pupil responses to visual events falling on the fovea compared to the periphery and the upper visual field compared to the lower visual field have been found in many studies (Strauch et al., 2022b). However, the exact relationship between the locations of visual events in the visual field and the amplitude of pupillary responses is more complicated. For instance, Thurman et al. (2021) found a context-dependent anisotropy (i.e. “blue sky effect”), showing an enhanced pupillary response to blue light in the upper regions of the visual field.

**Fig. 6:**
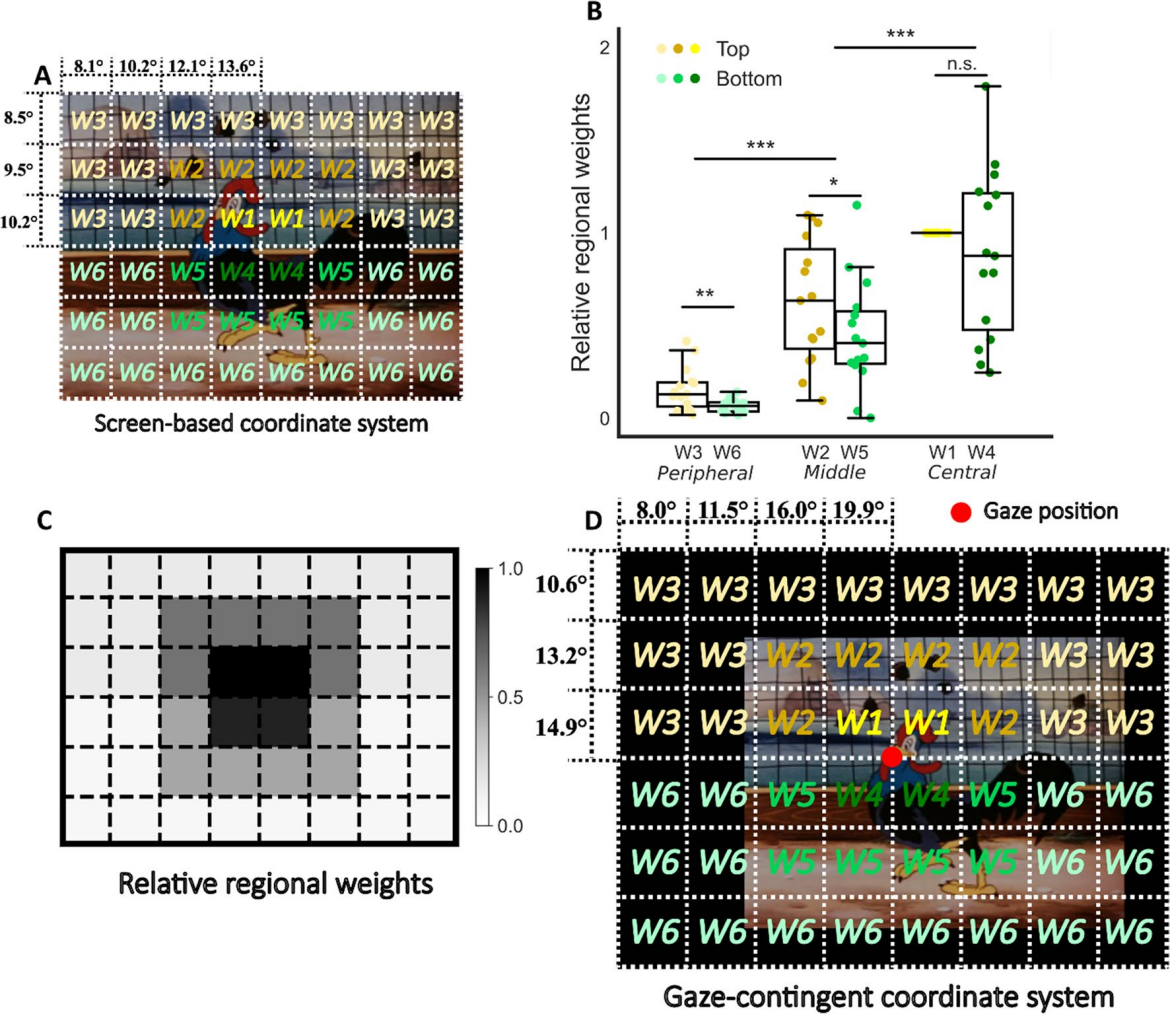
Regional weights of visual events across the visual field. **A** Regional weights and visual angles on the 48 regions. **B** Weights as fitted in the model, consistent with expected vertical and eccentric anisotropies. Each point represents a participant. **C** Illustration of the mapped regional weights averaged across participants. Darker means stronger weights. **D** Same illustration as in A but for the regional weights and visual angles of the gaze-contingent coordinate system. The *red point* represents the gaze position at this certain frame

To account for potential anisotropies, we split the visual field into 8 x 6 rectangular and equally sized regions (see Fig. 6A for visual degrees of each region). The number of regions was chosen to balance the trade-off between spatial resolution and computational power. Visual events were extracted for each region separately. Five free parameters were added as regional weights, accounting for relative contributions of the top-middle, top-peripheral, bottom-central, bottom-middle, and bottom-peripheral parts (see W1–W6 in Fig. 6A, the top-central weight (W1) was set to 1). Luminance and contrast changes per region were convolved with the RFs and then multiplied by the respective regional weights.

Incorporating regional weights significantly improved model performance (*R*^*2*^ = 0.302, *SD* = 0.085; *t*(14) = -5.425, *p* <0.001), comparing with the contrast response model. Weights selected by the model were consistent with expected visual field anisotropies: central image regions contributed more to the pupil dynamics than peripheral regions and so did top regions than bottom regions; see Fig. 6B, C). A two-way repeated-measures ANOVA showed significant main effects for the presence of vertical (top versus bottom; *F*(1,84) = 5.288, *p* = 0.024) and eccentric (central/middle/peripheral; *F*(2,84) = 73.268, *p* < 0.001) asymmetries, but no interaction (*F*(2,84) = 0.210, *p* = 0.811). Post hoc Turkey HSD tests showed significant differences in weights between peripheral and middle, and between middle and central regions (all *p* < 0.001).

#### Gaze contingency

Unrestricted eye movements would inherently result in a mismatch of the relative positions of the video image and the retina at each time point. For example, when the participant looks towards the top left (see Fig. 6D), the center of the video image no longer falls on the center of the retina. Furthermore, as most screens cannot completely cover the whole visual field of the participant, fixations adjacent to the screen's edges lead to more parafoveal stimulation by the background surrounding the monitor (see black bars surrounding movie images in Fig. 6D). We therefore incorporated gaze position in visual event extraction with a gaze-contingent (retinal) coordinate system representing the actual image falling upon the retina. To create this gaze-contingent coordinate system, a black rectangle 1.5 times larger than the size of the screen and with the same aspect ratio was created, representing the background surrounding the screen. The relative size of the background to the screen was chosen to ensure that the image of the screen stayed inside the borders of the background most of the time for relative peripheral fixations. Each image was then relocated in the gaze-contingent coordinate system by aligning the gaze position at the time of the frame to the center of the new coordinate system.

This resulted in a non-significant, but descriptive improvement (*R*^*2*^ = 0.327, *SD* = 0.087; *t*(14) = 1.92, *p* = 0.08), comparing with the regionally weighted model. Again, the selected regional weights supported the presence of anisotropies (foveal (central) > parafoveal (middle) > peripheral; top > bottom; see Supplementary Fig. 4C, D).

### Model evaluation and comparison

**Fig. 7:**
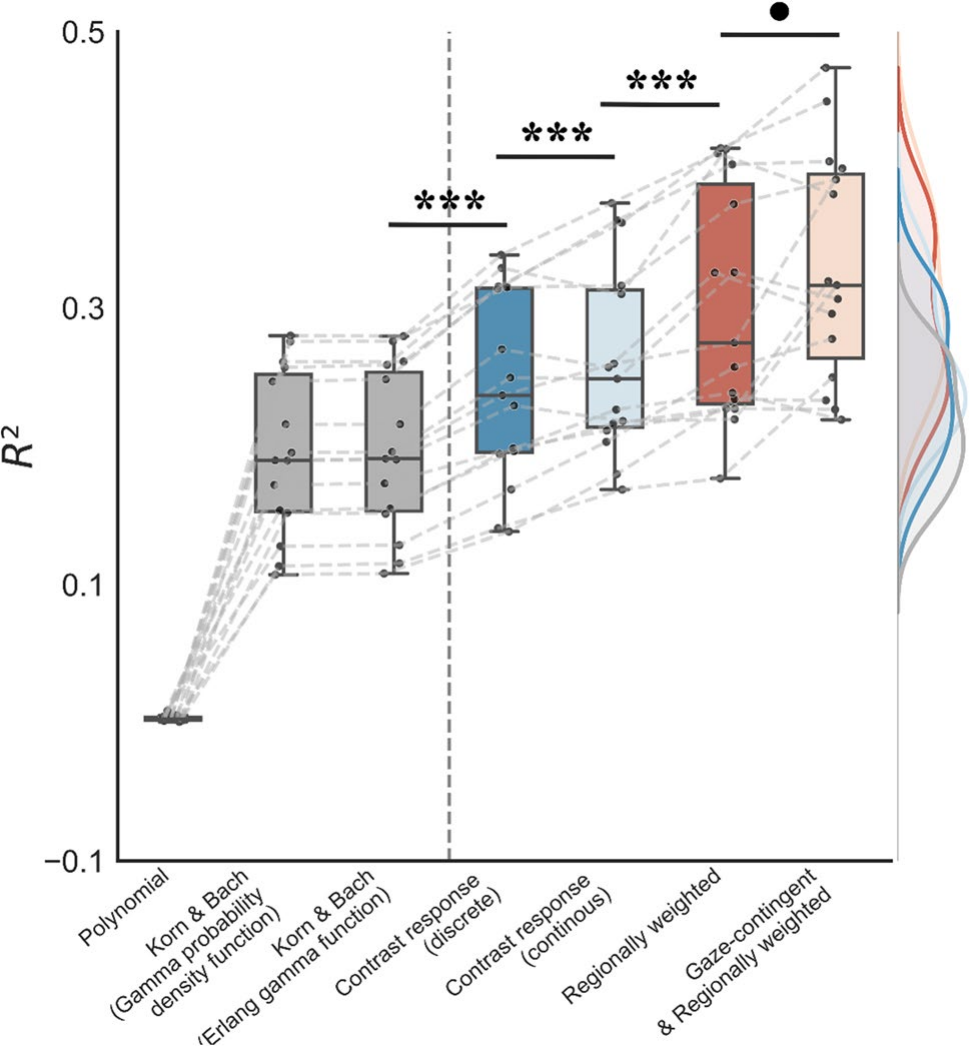
Explained variance per model. Boxplots show incremental improvements per modeling step. Single points, connected by dashed lines across models, illustrate model performance per participant. Shaded distributions on the right show smoothed density functions (histograms) of explained variance per model with corresponding colors (*** *p* < 0.001; • *p* = 0.08)

To evaluate model performance, we calculated *R*^*2*^ for each step in model development (see a summary of *R*^*2*^ for all steps in Fig. 7; see Supplementary Table 1 also for correlation coefficients and RMSE). Gaze-contingency model incorporating all described steps outperformed all other models. To further compare models, the Bayesian information criterion (BIC) provides insights by considering parsimony (i.e., explained variance relative to model complexity set by the number of free parameters). Note that BIC could only be meaningfully calculated for the later three models, as the split of training and testing data (i.e., cross-validation) resulted in a different sample size relative to the discrete event-related fitting approach. The decreasing BIC values over the last three models (see Supplementary Table 1) suggested meaningfully improved model performance despite higher complexity. In summary, we develop Open-DPSM with a “temporally continuous modeling” for better prediction of pupil size change to our dynamic and complex stimuli and we improve model predictions by incorporating (i) a scaled contrast response function instead of an undershoot-correction function, (ii) regional weights, and (iii) gaze-contingent coordinate system.

To replicate our findings, we further evaluated model performance on a different dataset (Gestefeld et al., 2020). Here, the movies used in our experiment were tested together with many other clips of similar length with a different group of participants and a different lab with a smaller monitor. The pattern of results fell in line with the findings reported above (see Supplementary Material “Replication of findings with different data (Gestefeld et al., 2020)” for details).

## Python package (*Open-DPSM.py*)

**Fig. 8:**
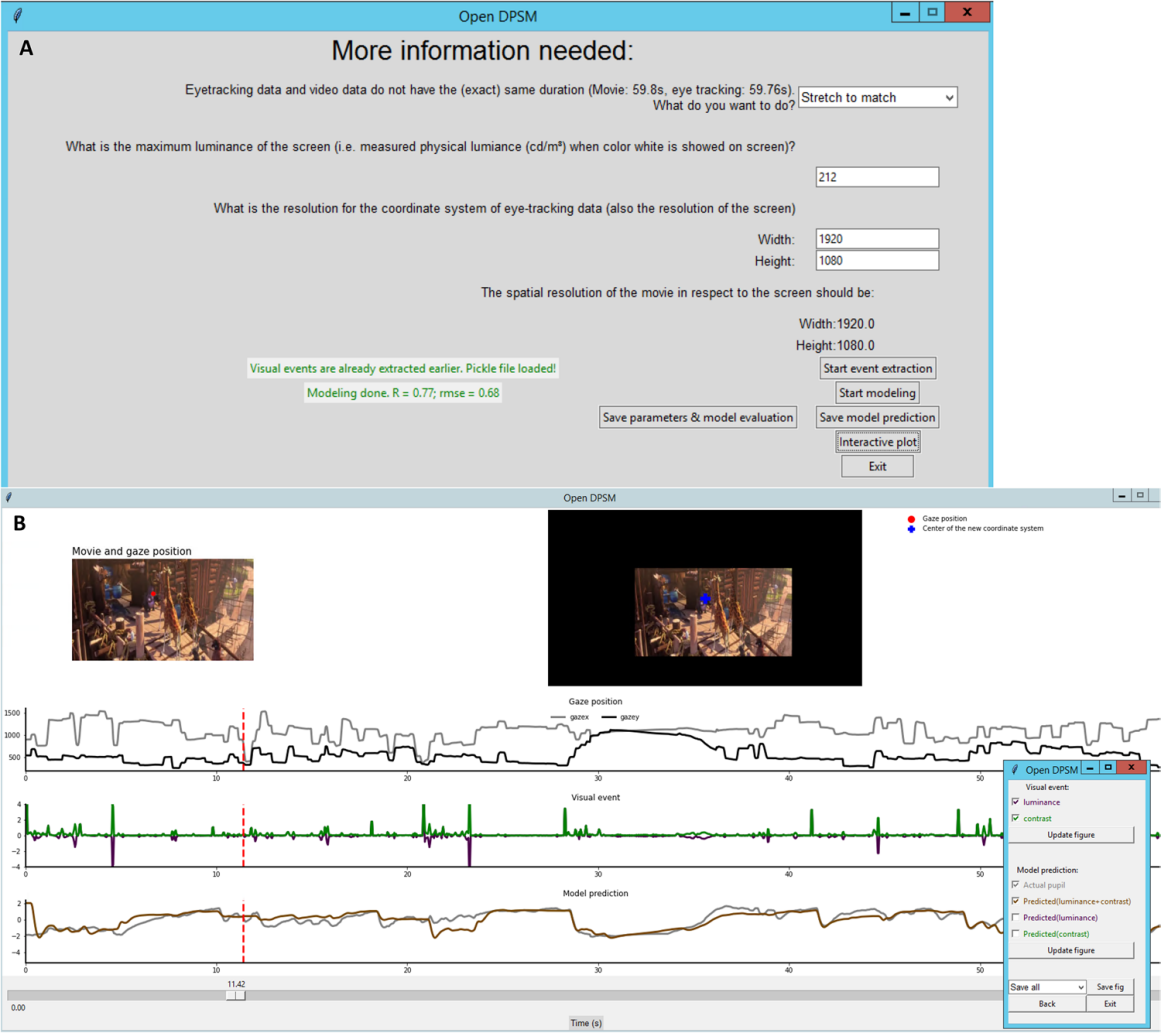
Screenshots of the Open-DPSM GUI. **A** Screenshot of the GUI page of Visual event extraction & pupil prediction. **B** Screenshot of the interactive plotting GUI. To start the GUI, run *main_app.py*. Video (and optional eye-tracking data) are loaded on the welcome page (not depicted), and relevant context information should be entered by the users in the GUI shown in panel A. The main functions, *events_extraction()* and *pupil_prediction()* can be called via the buttons shown in panel A. Model performance is outputted on the left side of the screen. A click on “interactive plot” opens the screen shown in panel B with the data traces of gaze positions, extracted visual events, and both predicted and observed pupil size over time. Dragging the slider, shown at the bottom of panel B, allows users to jump to a specific time point and video frame

Open-DPSM can be downloaded via https://github.com/caiyuqing/Open-DPSM. It has been tested on Microsoft Windows 2012 R2 with Spyder (version 5), Jupiter notebook (6.4.5)/JupiterLab (3.2.1) and PyCharm (2013.1.4) installed that used Python version 3.9.7. The main Open-DPSM script (*main.py*) contains two classes of functions to perform visual event extraction (*event_extraction.py*) and pupil prediction (*pupil_prediction.py*) as well as one class for interactive plotting of results (*interactive_plot.py*). Another script (*settings.py*) allows to set default parameters (e.g., number of image regions, size of the gaze-contingent coordinate system, gamma value of the monitor, etc.), which can be adjusted by experienced users. For users who prefer a graphical user interface (GUI), the script *main_app.py* activates the Open-DPSM GUI, which can conduct all modeling steps in a user-friendly manner (Fig. 8).

### classes.event_extraction

This class of functions serves to extract the timing and strength of visual events (luminance and contrast changes) of an input video file. This trace is needed later for convolution to make a pupil prediction. The function *event_extraction()* extracts visual events (e.g., luminance changes) frame by frame from video files (.mp4, .avi, .mkv, .wmv, .mov, .flv, .webm) per image region. When eye tracking data file is provided (.csv) with timestamps and gaze coordinates, gaze-contingent visual events are extracted. Gaze data is automatically downsampled to the video sampling rate. If no eye-tracking data file is provided, screen-based visual events are extracted instead. The function outputs video information (resolution, frame rate, etc.), timestamps, and event traces per image region, which will be saved in a pickle file named “[movieName]_[subjectName]_VF_LAB_6X8.pickle” in a “Visual events” folder.

### classes.pupil_prediction

This class of functions serves to model pupil size from the extracted visual events by fitting two response functions, convolved with the extracted visual events from *event_extraction()*. Without eye-tracking data, a predicted pupil trace is generated based on the average pupil response functions calculated from the data described in this manuscript (see “Integration of a contrast response function”). When eye-tracking data is available, model optimization will be performed and best-fitting parameters for response functions and regional weights will be found and saved in a “csv results” folder as a .csv file named “[movieName]_[subjectName]_parameters.csv” once finished. The results of model performance will also be saved in the same file (correlation and RMSE). The file contains two columns, with the names of the parameters and their values, respectively. Actual and predicted pupil size based on both, as well as combined, visual event traces are saved in another .csv file in the same folder named “[movieName]_[subjectName]_modelPrediction.csv”, which will contain actual pupil size and predicted pupil size as columns. Predicted pupil size (z-standardized) will be provided with three columns, one for the combined prediction with both luminance and contrast change, one for prediction with luminance change only, and one for prediction with contrast change only.

### classes.interacitve_plot

This class of functions can be used to interactively plot results of *event_extraction()* and *pupil_prediction()*. *interactive_plot()* produces three subplots as in Fig. 8B, one each for gaze position, visual events extracted via *event_extraction()*, and the trace of predicted and actual pupil size changes. Subplots can be saved as individual files using the interface. Specific frames can be selected using a slider.

## Discussion

Pupil size changes are the integrated outcome of several underlying factors, encompassing effects of changes in a range of low-level sensory features, but also higher-order cognition. To isolate the cognitive effects from the sensory effects, many studies have opted for quantifying the expected influence of the pupil light response (PLR), a primary contributor to pupil size change. Thus far, only a few studies have adopted dynamic models of PLR to estimate pupil size change in presenting complex dynamic stimuli (Fanourakis & Chanel, 2022; Napieralski & Rynkiewicz, 2019). However, those studies did not consider other low-level features, beyond luminance, and disregarded visual field anisotropies or coarsely controlled for these by exclusively focusing on visual events falling on foveal regions.

We here introduce Open-DPSM, an open-source toolkit that enables not only the prediction of pupil size with dynamically changing stimulus materials but also the extraction of visual events from videos. What are Open-DPSM’s key features? (i) Open-DPSM uses a parsimonious convolutional method with simple gamma functions to model pupil responses; (ii) Open-DPSM models pupillary dynamics more accurately by incorporating a separate transient for orienting responses that scale with contrast changes; (iii) Open-DPSM builds on a realistic representation of visual input to pupil size changes by weighing the relative contribution of visual events across the visual field contingent to gaze positions – and incorporates the cortical magnification factor of foveal input. (iv) Open-DPSM is a publicly available tool that provides accessible and open-source Python functions for modeling pupil size while offering high flexibility for future adaptations.

### Theoretical implications

The contrast response model demonstrates that pupil responses to changes in luminance reflect more than just a simple change to light. Korn and Bach (2016) highlighted the importance of modeling pupil size changes with a dual-component model, and we here demonstrate that a pupil response is a combination of the responses to light (dilations to dark events, constrictions to bright events) and contrast (constrictions that scale with contrast). This second component is therefore beyond a phenomenological ‘overshoot’ of a pupil light response, but part of an orienting response to salient (high contrast) events (Barbur et al., 1992; Gamlin et al., 1998; Hu et al., 2019; Kanari & Kaneko, 2021; Naber et al., 2011; Nakano et al., 2021; Slooter & van Norren, 1980; Young et al., 1995; for a review, see Strauch et al., 2022a), which is likely modulated by attention (Koevoet et al., 2023; Naber et al., 2013; Strauch et al., 2022b).

### Future work and model extensions

Open-DPSM is designed for seamless integrations of additional event streams to achieve higher modeling performance. Whilst the current model only took contrast into account, further low-level visual features, such as changes in color (Barbur et al., 1992), spatial frequency (Barbur & Thomson, 1987; Young et al., 1995), and orientation (Hu et al., 2019) can be incorporated easily. This opens up new avenues for studying low-level visual features with dynamic stimuli – allowing for the decomposition of contributing factors. Further improvements and insights are plausible here, as such features are characterized by distinct response properties (Young et al., 1993; Young & Kennish, 1993). Furthermore, different transformations of low-level features, such as Michelson Contrast (Michelson, 1927), may result in an improved model of pupillary changes (Sandoval Salinas et al., 2020; Wang et al., 2014). As illustrated in previous work (e.g. de Gee et al., 2014; Denison et al., 2020; Korn & Bach, 2016; Lempert et al., 2015; Willems et al., 2015), the same convolutional approach can also be used to model cognitive events. Open-DPSM should, in principle, be applied to model cognitive events given an appropriate event trace, but further evaluation is required.

Our data support the finding that pupil responses to luminance changes are stronger in the central and top than in the peripheral and bottom parts of the visual field (Istiqomah et al., 2022; Strauch et al., 2022b). It is worth mentioning that the current rectangular grid, which evenly divides the visual fields into subregions is a first, but coarse, representation of the visual field. Future versions of Open DPSM should, however, model the visual field as an elliptical (or distorted elliptical) shape (Anderson et al., 2014; Baldwin et al., 2012; Engel, 1977) and take into account the cortical magnification factor. Moreover, the current model also simplified anisotropies by applying the same set of regional weights to both luminance and contrast responses. Different asymmetries across the visual field have been described for a range of visual features (Thurman et al., 2021). Such models could also be used to study relative regional contributions of low-level features to the pupil light response, but also attention and vision more broadly.

In addition, many factors that contribute to (estimated) pupil size changes have not yet fully been explored. For instance, distortions of estimated pupil size resulting from changing angles between camera and eye as the eyes move should be accounted for using foreshortening error corrections (Gagl et al., 2011; Hayes & Petrov, 2016; see Korn et al., 2017 for an implementation). Other factors such as pupil size change before and during saccades (Koevoet et al., 2023; Wang et al., 2015), pupil responses to different durations or temporal frequencies of changes in visual features, pupil responses to depth (pupil near response; Pielage et al., 2022), and pupil responses to changes in other sensory domains (e.g., body movements and audition; Van der Stoep et al., 2021) are also expected to influence pupil size changes, which will need to be incorporated in future versions of the current model to explain even more variance in pupil dynamics.

### Use cases of Open-DPSM

Pupillometry has widespread applications in research and practice (see Binda & Gamlin, 2017; Einhäuser, 2017; Mathôt, 2018; Strauch et al., 2022a for reviews). How can Open-DPSM help researchers and practitioners alike? In **neurology/ophthalmology**, pupil light responses serve as an objective indicator for the dysfunction of vision. Open-DPSM is especially promising for the diagnosis of spatial-attentional disorders such as visual field defects and hemispatial neglect (Lasaponara et al., 2021; Naber et al., 2018; Portengen et al., 2023b; Ten Brink et al., 2023), as it can map the responsivity of the pupil across space, which is, in turn, indicative of visuoattentional deficits. This would be particularly beneficial for perimetric testing of special populations such as children and brain-injured patients, as testing could be done highly automatized and with engaging/naturalistic stimuli, and substantial data can be collected within a short period (Gestefeld et al., 2020, 2021). The application may also extend to the measurement of the sensitivity of other low-level features, such as contrast sensitivity (Hernández et al., 1996; Slooter, 1981; Slooter & van Norren, 1980). In **psychology/affective and cognitive neuroscience** (but also beyond), pupil dilation is used to study cognitive processes and respective neural underpinnings, such as the effects of mental effort (McLaughlin & Van Engen, 2020; Van Der Meer et al., 2010; Zekveld & Kramer, 2014), emotional processing or regulation (Bradley et al., 2008; Henderson et al., 2014; Kinner et al., 2017; Koevoet et al., 2023; Võ et al., 2008; Wetzel et al., 2020; Zimmermann & Bach, 2020) and attention (Binda et al., 2014; Denison et al., 2020; Naber et al., 2013). Thus far, stimuli highly controlled for low-level features are usually adopted to prevent confounding effects of those features. Open-DPSM can provide an estimate of expected pupil size changes to stimulus material. Previous studies have shown that despite maintaining constant global luminance, variations in local regions of the stimuli can still exert an influence on pupil size, such as luminance differences in the face areas (Laeng et al., 2018) or relative size of bright versus dark regions in the eye (Derksen et al., 2018). Open-DPSM can generate predictions of how pupil size would change with given stimuli and thereby enable researchers to estimate the variance that can be attributed to the stimuli themselves. This would facilitate the examination of potential confounds of low-level design issues in pupillometric studies. Furthermore, Open-DPSM accommodates complex and dynamically changing stimuli as input material to leverage the high sensitivity of pupillometry to cognitive processes – in the lab settings or in real world applications. Previous studies using dynamic stimuli, such as movie-watching (Raiturkar et al., 2016; Soleymani et al., 2012), video-gaming (Fanourakis & Chanel, 2022; Mitre-Hernandez et al., 2021), or driving (Kerautret et al., 2021; Pedrotti et al., 2014; Vintila et al., 2017), adopted different methods to compensate for the effects of luminance. It has been found that modeling the PLR is more advantageous in the sense that it quantifies the effectiveness of separating effects reflected in the pupil (Fanourakis & Chanel, 2022; Wong et al., 2020). In addition, Open-DPSM provides estimations of temporal properties for pupil responses, such as latency and timing of peak amplitude (Denison et al., 2020), which are especially important for the investigation of cognitive events.

## Conclusion

We introduce Open-DPSM, an openly available toolkit that offers a robust and flexible approach to model pupil size changes to dynamic visual stimuli. Its incorporation of multiple visual events, regional weights, and gaze-contingent visual event extraction provides new insights into the effects of visual input on pupil size and visual processing and can pave the way for assessing and using attentionally modulated pupil size changes outside highly constrained lab settings. The availability of Open-DPSM as an open-source package enhances its accessibility and potential for further advancements in many fields of research.

### Supplementary material

ESM 1 Find the supplementary material via https://osf.io/qvn64/. (DOCX 1860 kb)

## Data Availability

All data are available via https://osf.io/qvn64/. As our stimuli are a subset of stimuli from Gestefeld et al. (2020), experimental materials can be downloaded directly from their open dataset: https://dataverse.nl/dataset.xhtml;jsessionid=f84f78bc461ba0a539430ae788cd?persistentId=doi%3A10.34894%2FLEYVL8&version=&q=&fileTypeGroupFacet=%22Document%22&fileAccess=&fileSortField=type

## Footnotes

1 To avoid local minima in fitting, we also tested the “basinhopping” function from the same package with 20 iterations. Model performance was similar, but the fitting time increased significantly. Hence, all the reported results used “minimizing” with a fixed starting point. Both options are provided in the toolbox.

## Declarations

### Preregistration

The experiment was not preregistered.
