# Supplementary material for: Open-DPSM: An open-source toolkit for modeling pupil size changes to dynamic visual inputs
Source: Behav Res Methods. 2023 Dec 11;56(6):5605–21. doi: 10.3758/s13428-023-02292-1 (PMC11335788; doi:10.3758/s13428-023-02292-1)

# Supplementary Material to “Open-DPSM: An open-source toolkit for modeling pupil size changes to dynamic visual inputs”

Supplementary Table 1

Summary of model performance

|  |  | *R* | *R^2^* | RMSE | BIC |
| --- | --- | --- | --- | --- | --- |
| Korn & Bach (2016) | RF1 | 0.438 | 0.196 | 1.059 |  |
|  | RF2 | 0.438 | 0.196 | 1.058 |  |
| Contrast response model (temporally discrete) | RF1 | 0.487 | 0.242 | 1.010 |  |
|  | RF2 | 0.487 | 0.242 | 1.010 |  |
| Contrast response model (temporally continuous) | RF1 | 0.505 | 0.259 | 0.992 | -164.433 |
|  | RF2 | 0.507 | 0.261 | 0.990 | -201.687 |
| Regionally weighted model | RF1 | 0.543 | 0.300 | 0.952 | -877.005 |
|  | RF2 | 0.544 | 0.302 | 0.950 | -904.221 |
| Gaze-contingent model | RF1 | 0.569 | 0.328 | 0.923 | -1423.287 |
|  | RF2 | 0.567 | 0.327 | 0.925 | -1391.486 |

*Note.* RF1: Gamma probability density function; RF2: Erlang gamma function

Supplementary Table 2

Binned luminance change for Contrast response model

|  | Change in brightness | Mean (cd/m2) | Range (cd/m2) |
| --- | --- | --- | --- |
| Bin1 (0-20 percentile) | Very dark | -20.8 | -45.8~-10.4 |
| Bin2 (20-40 percentile) | Slightly dark | -5.3 | -9.9~-3.2 |
| Bin3 (40-60 percentile) | Very small or none | 1.1 | -3.2~2.8 |
| Bin4 (40-60 percentile) | Slightly bright | 4.3 | 2.9~6.9 |
| Bin5 (40-60 percentile) | Very bright change | 17.7 | 7.2~46.3 |

Supplementary Fig. 1

*Relationship between luminance changes and pupillary response amplitudes in movies*


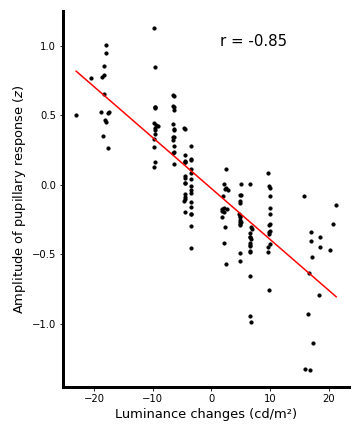


*Note*. An illustration of the linear relationship between Luminance changes (cd/m2) (in 10 bins) and pupillary response amplitudes (constrictions) within current limited range of luminance changes in movies

Supplementary Fig. 2

*Simulated result of Illuminance Flash Task with Contrast Response Model*


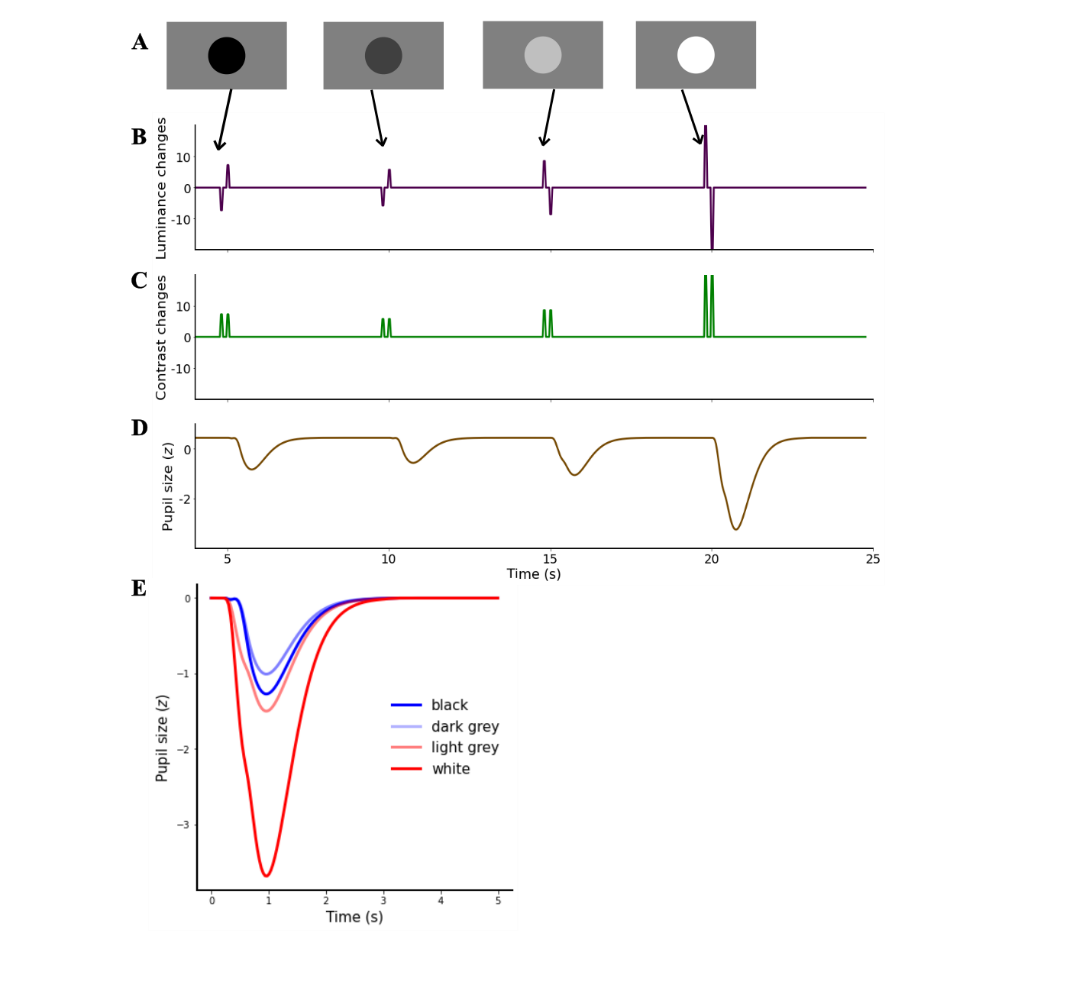


*Note*. A We simulated the illuminance flashes task *(Korn & Bach, 2016)* as a movie for input to our model. In the movie four circles (black, dark grey, light grey and white) appear at the center of a grey background for 200ms. We set ITI to 5s such that circles appear at 5s, 10s, 15s and 20s. Then we extracted the luminance B and contrast C changes from this movie. Next, *we* convolved these feature changes with two response functions (Luminance: *n* = 9.67, *t_max_* = 0.19s; Contrast: *n* =3, *t_max_* = 0.62s) and combined the two predictions with a relatively stronger weight for contrast than luminance (3.6 to 1). D The resulting fit showed that, like in Korn & Bach (2016), our contrast response model can also demonstrate the counterintuitive observation of constriction response to darkness, as well as the asymmetric delay. Importantly, the asymmetric delay in our case emerged from the mitigation of the dilation response to luminance increases and the enhancement of the constriction response to contrast changes (for the contrast response component per luminance condition). These responses cancel each other out in the first milliseconds, explaining a delayed pupil response. E Constriction responses in D with the appearance of the circle as the start.

Supplementary Fig. 3

All response functions selected by different models


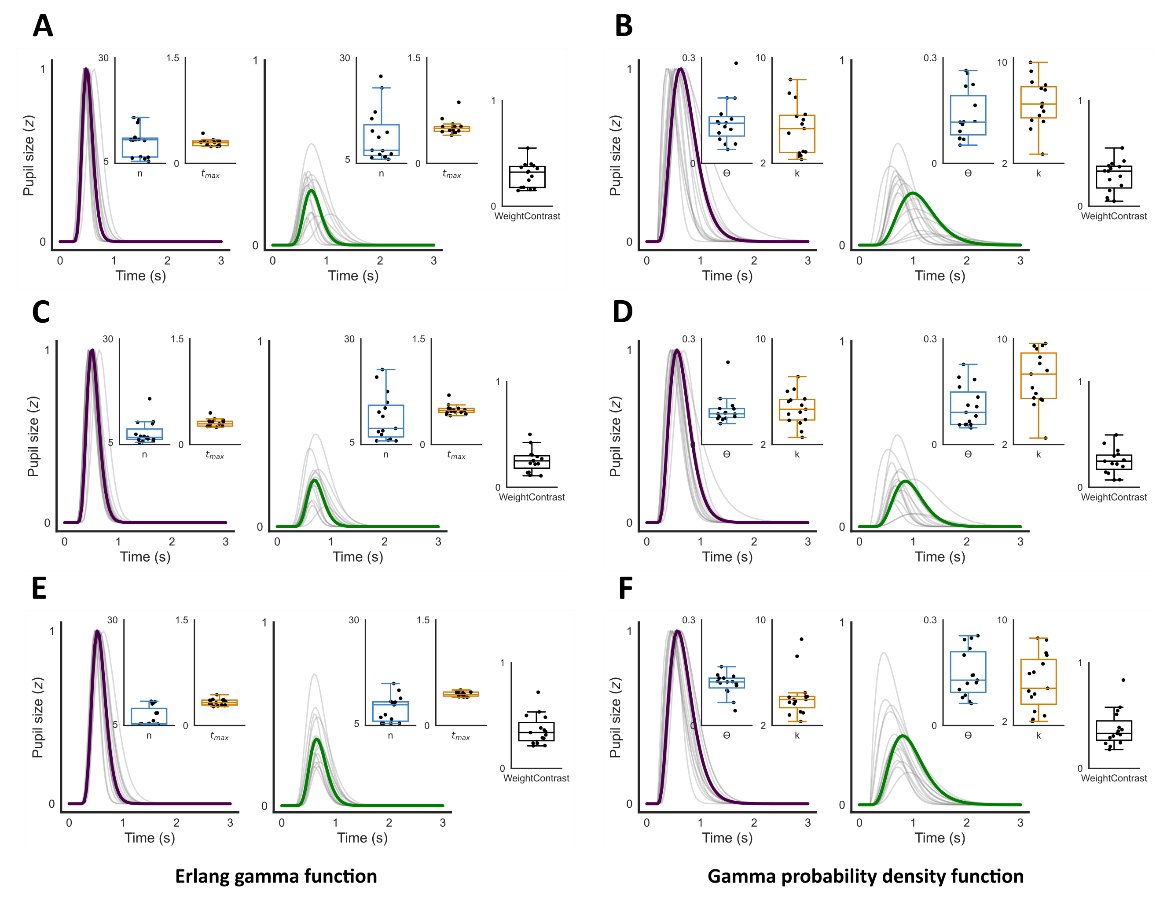


Note. **A & B** Response functions selected by Contrast response model. **C & D** Response functions selected by Regionally weighted model. **E & F** Response functions selected by Gaze-contingent model. The Left column illustrates results for the Erlang gamma function (A, C, E), and the right column illustrates results for the Gamma probability density function (B, D, F). The purple lines are the average of luminance response functions and the green lines are the average of contrast response functions for all participants (grey lines: RFs per participant). The box plots at the top right of each subplot illustrate selected parameters (dots represent individual participants). As each response function contains two free parameters, they are represented by two boxplots separately (n (blue) and t_max_ (orange) for Erlang gamma function; Θ (blue) and k (orange) for gamma probability density function). The weight of the contrast RF, representing the relative contribution of the contrast response relative to the response to luminance, is plotted in the third boxplot (black).

Supplementary Fig. 4

Illustration of the regional weights selected by the models


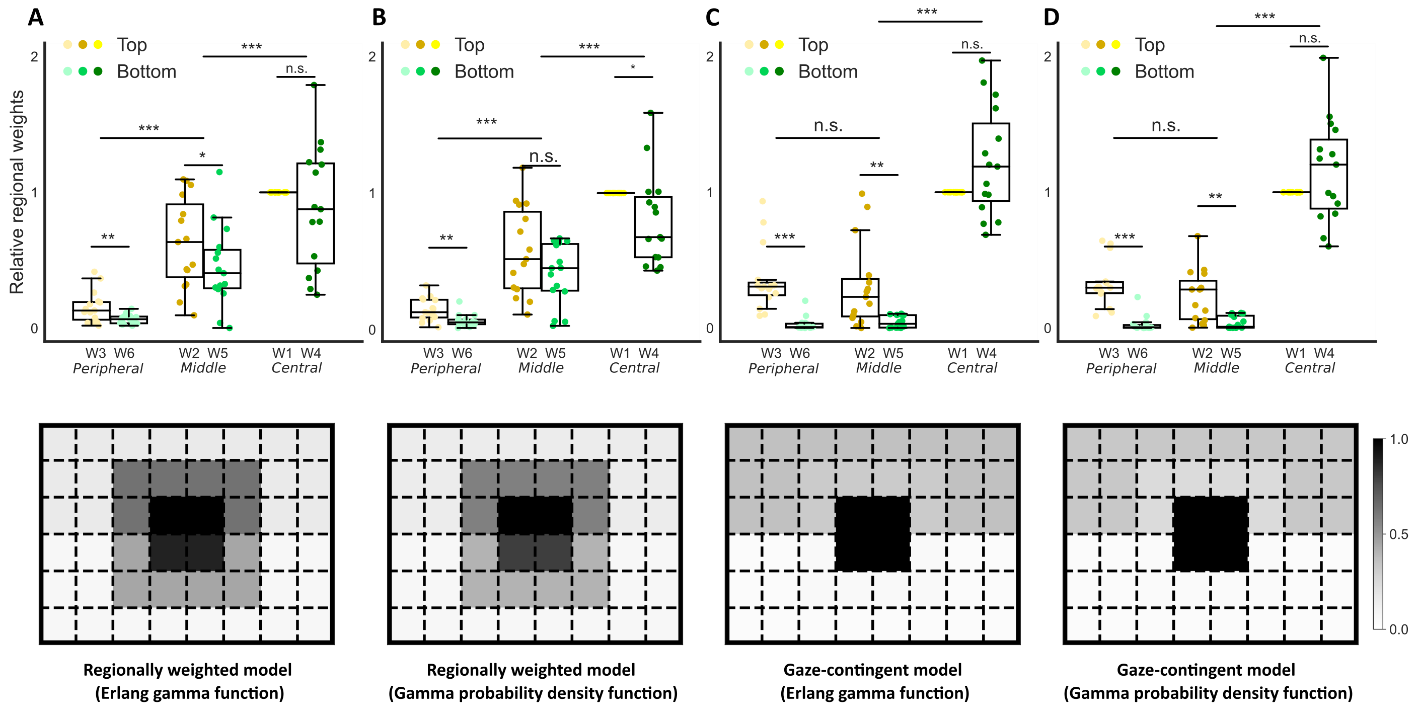


Note. **A & B** Regional weights selected by Regionally weighted model. **C & D** Regional weights selected Gaze-contingent model. A & C adopted Erlang gamma function and B & D adopted Gamma probability density function (A is the same plot as Fig. 6 C & D). See Fig. 6 for details.

# Replication of findings with a different dataset (Gestefeld et al. 2020)

To make sure that the results can be generalized to other apparatus/movies, a dataset collected by Gestefeld et al. (2020) was used for replication.

## Data

### Participants & stimulus material

The dataset contains 40 participants. Note that we here only used data from *n*= 33 participants with sufficient data quality and only those trials in which the whole visual field was non-occluded.

All participants freely watched 19 to 74 (*M*= 43.0, *SD*= 26.7) movie clips of 60 s each. Of these, 18.4% were presented together with audio on the left ear, 18.3% on the right ear, 20.5% on both ears and 42.8% without audio (see Gestefeld et al. (2020) for more details).

### Apparatus

Movies were presented on a LaCie CRT monitor positioned at 60 cm from the eye with a size of 40*30 cm (1152 x 870 pixel; 36.9° x 28.1° visual angle). The maximum 100% brightness of the TV was 280 cd/m^2^ with a gamma value of 2.2. Gaze position and pupil size were tracked with an Eyelink 1000 (SR Research, Ontario, Canada) at 500 Hz, and a chin- and forehead rest was used.

## Results

All steps were the same as in the main manuscript. Results are reported in the following (see Supplementary Table 3 for an overview of all the model performance for this dataset).

Supplementary Table 3

Summary of model performance for Gestefeld et al. (2020) data

|  |  | *R* | *R^2^* | RMSE | BIC |
| --- | --- | --- | --- | --- | --- |
| Korn & Bach (2016) | RF1 | 0.509 | 0.265 | 0.988 |  |
|  | RF2 | 0.495 | 0.251 | 1.002 |  |
| Contrast response model (temporally discrete) | RF1 | 0.550 | 0.308 | 0.945 |  |
|  | RF2 | 0.547 | 0.305 | 0.948 |  |
| Contrast response model (temporally continuous) | RF1 | 0.544 | 0.303 | 0.949 | -2616.964 |
|  | RF2 | 0.543 | 0.302 | 0.950 | -2575.881 |
| Regionally weighted model | RF1 | 0.555 | 0.315 | 0.938 | -3200.392 |
|  | RF2 | 0.553 | 0.314 | 0.939 | -3122.452 |
| Gaze-contingent model | RF1 | 0.570 | 0.331 | 0.921 | -3868.666 |
|  | RF2 | 0.569 | 0.331 | 0.922 | -3821.143 |

*Note.* RF1: Gamma probability density function; RF2: Erlang gamma function

### Benchmark models

#### Polynomial model. The performance was poor (*R^2^* < 0.001).

#### Extended Korn & Bach (2016) model. Two response functions (RFs) with three free parameters were fitted (RF in LTI1 for dilation: *c* =0.03, *k* =13.1, *Θ* = 0.03, supplementary Fig. 3B; RF in LTI2 for the difference between dilation and constriction: *c* = 2.26, *k* = 2.61, *Θ* = 0.35; supplementary Fig. 3C), as was the weight for the second RF (*weight* = 1.002). The weight for the first RF was set as 1. This model explained almost all variation in both dilation (*R^2^* = 0.98), and a large part of the variation for the constriction (*R^2^* = 0.89).

When applying the two RFs to the time-series pupil data, the two RFs explained the variation in pupil substantially above chance, but much less than for the separated event-related responses (*R^2^* = 0.265) and the RF of the LTI1 contributed much more strongly to explaining variance (*R^2^* = 0.237) than LTI2 (*R^2^* = 0.081).

### Open-DPSM

#### **Integration of contrast response function.**

#### **Modeling results.** After integrating the scaled contrast response into the model, the model improved significantly (*R^2^* = 0.305, *SD* = 0.076; *t*(32) = -9.82, *p* < 0.001; best-fitting parameters: RF for luminance change: *n* = 9.5, *t_max_* = 0.23; RF for contrast change: *n* = 1.0, *t_max_* = 0.57; weight for contrast change = 0.37). See Supplementary Table 4 for the average and range of luminance changes per bin.

Supplementary Table 4

Binned luminance change for Contrast response model in Gestefeld et al. (2020) data

|  |  | Mean (cd/m^2^) | Range (cd/m^2^) |
| --- | --- | --- | --- |
| Bin1 (0-20 percentile) | Very dark change | -27.55 | -56.96~-13.35 |
| Bin2 (20-40 percentile) | Slightly dark change | -7.50 | -13.19~-4.12 |
| Bin3 (40-60 percentile) | Very low change or no change | -0.41 | -4.10~3.70 |
| Bin4 (40-60 percentile) | Slightly bright change | 6.43 | 3.17~11.06 |
| Bin5 (40-60 percentile) | Very bright change | 24.69 | 11.21~55.57 |

##### Convolving RFs directly with luminance and contrast changes. With the temporally continuous modeling method, the model performance (R^2^ = 0.302, SD = 0.086) was compatible with the temporally discrete modeling method (t(32) = -0.684; p = 0.499). A comparison of all the RFs for all the participants can be found in Supplementary Fig. 5.

Supplementary Fig.5

All response functions selected by different models in Gestefeld et al. (2020) data

***
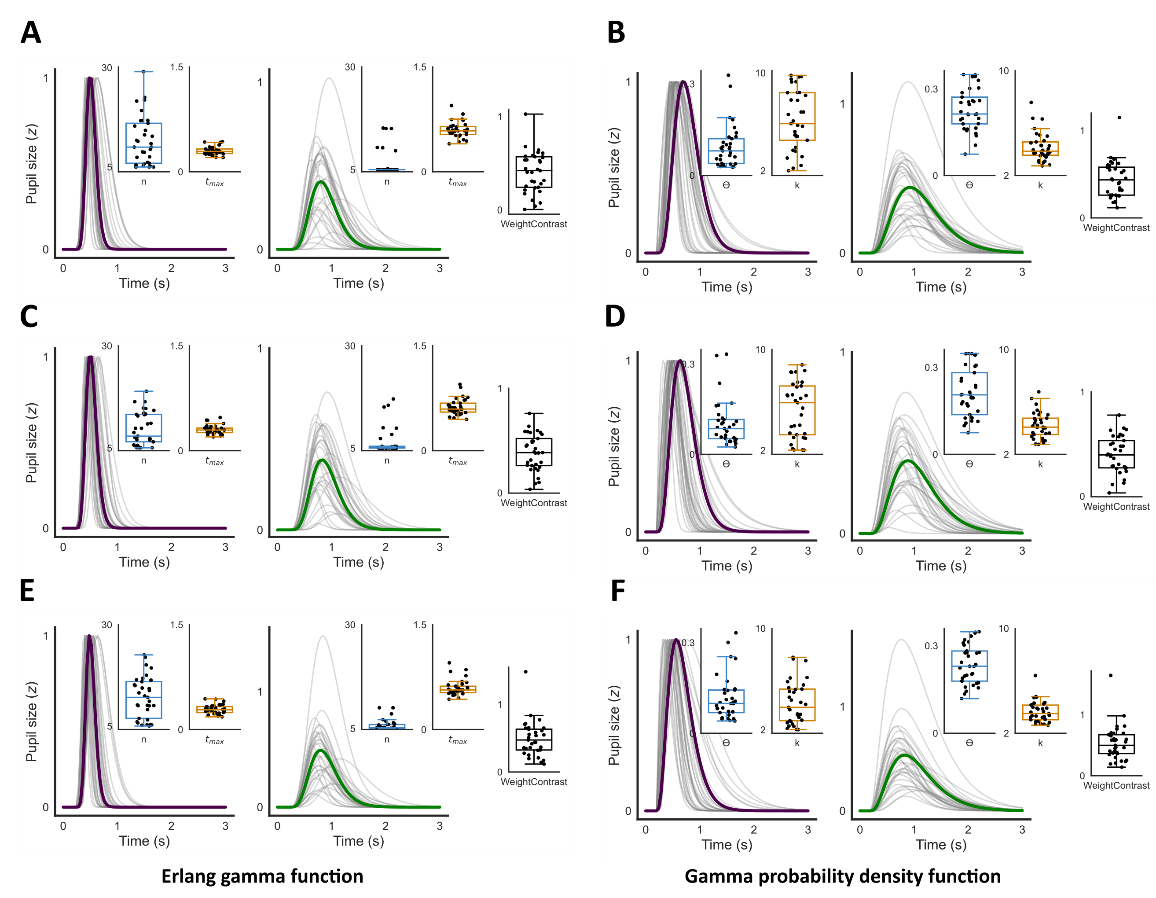
***

*Note.* **A & B** Response functions selected by Contrast response model. **C & D** Response functions selected by Regionally weighted model. **E & F** Response functions selected by Gaze-contingent model. The Left column illustrates results for the Erlang gamma function (A, C, E), and the right column illustrates results for the Gamma probability density function (B, D, F). See Supplementary Fig. 1 for more information.

#### Regionally weighted. The model improved significantly (*R^2^* = 0.314, *SD* = 0.093; *t*(32) = -3.300, *p* = 0.002). Similar visual field anisotropies as in the main manuscript were found (center > middle > peripheral; Upper > lower). A two-way repeated measures ANOVA showed significant main effects for the presence of vertical (top versus bottom) (*F*(1,192) =27.011, *p* <0.001) and eccentric (Central/Middle/Peripheral) (*F*(2,192) =23.659, *p* <0.001) asymmetries but no interaction (*F*(2,192) = 0.861, *p* =0.424). Post-hoc Turkey HSD tests demonstrated a significant difference for weights selected between peripheral and middle (p<0.001), but no significant difference between middle and central regions (*p* = 0.775) (Supplementary Fig.4 A & B).

**Supplementary Fig. 6**

Illustration of regional weights selected by the models in Gestefeld et al. (2020) data

***
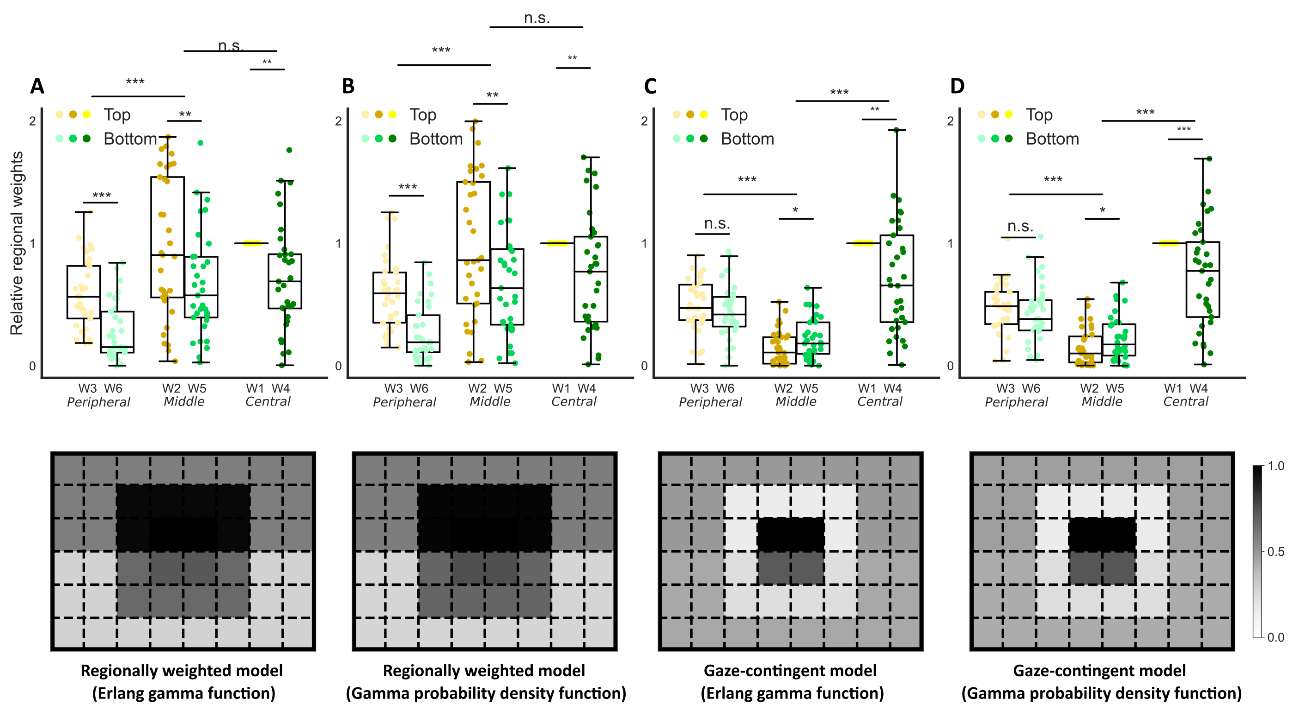
***

*Note*. **A & B** Regional weights selected by Regionally weighting model. **C & D** Regional weights selected Gaze-contingency model. A & C adopted Erlang gamma function and B & D adopted Gamma probability density function. See Fig. 6 for details.

#### Gaze contingency. With the gaze-contingent visual events, the model performance improved significantly compared with the screen-based coordinate system (*R^2^*= 0.331, *SD* = 0.087; *t*(32) = 5.018, *p* <0.001). Moreover, regional weights selected by the model showed anisotropies. However, the direction of the eccentricity effect was not consistent with the findings in the main article, as both weights in peripheral and central (or foveal) regions were higher than in the middle (or parafoveal) regions (Supplementary Fig. 4 C & D). Note that visual angles and physical sizes for the regions were quite different between Gestefeld et al (2020) data and our data used in the main text (see Fig.6 and supplementary Fig. 5). Hence, results on regional weights were not directly comparable.

***Supplementary Fig. 7***

Regional weights and visual angles in Gestefeld et al. (2020) data.


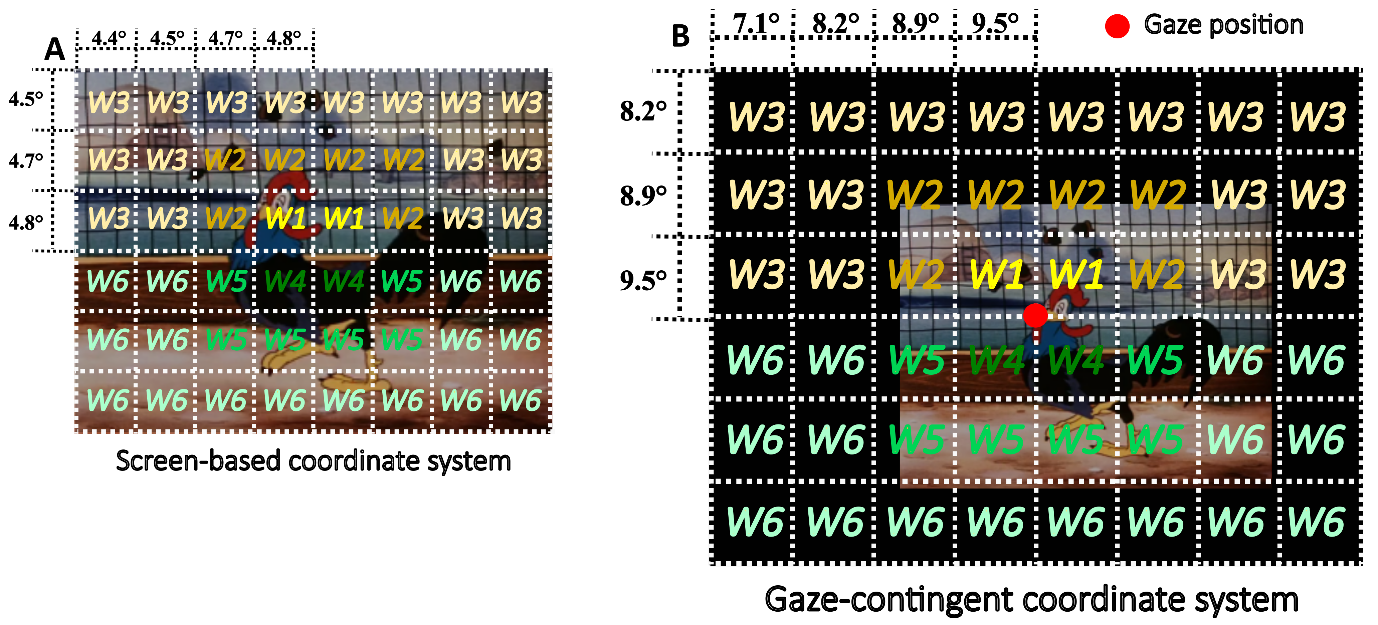


*Note*. **A** An illustration of the weights and visual angles on the forty-eight regions used in the regionally weighted model in the Gestefeld et al. (2020) dataset. **B** Same illustration for gaze-contingent model. Redpoint represents the gaze position at this certain frame.

## Model comparison and evaluation

Overall, the results of Gestefeld et al. (2020) data were very similar to the results of our dataset (Supplementary Table 3 and Supplementary Fig. 6) and hence, replicated the main findings of the current study. The final temporally continuous model with all described procedures, including integrating responses to contrast change, adding regional weights and adopting gaze-contingent visual event extraction, outperformed all other models.

Supplementary Fig. 8

R^2^ for all models for Gestefeld et al. (2020) dataset.


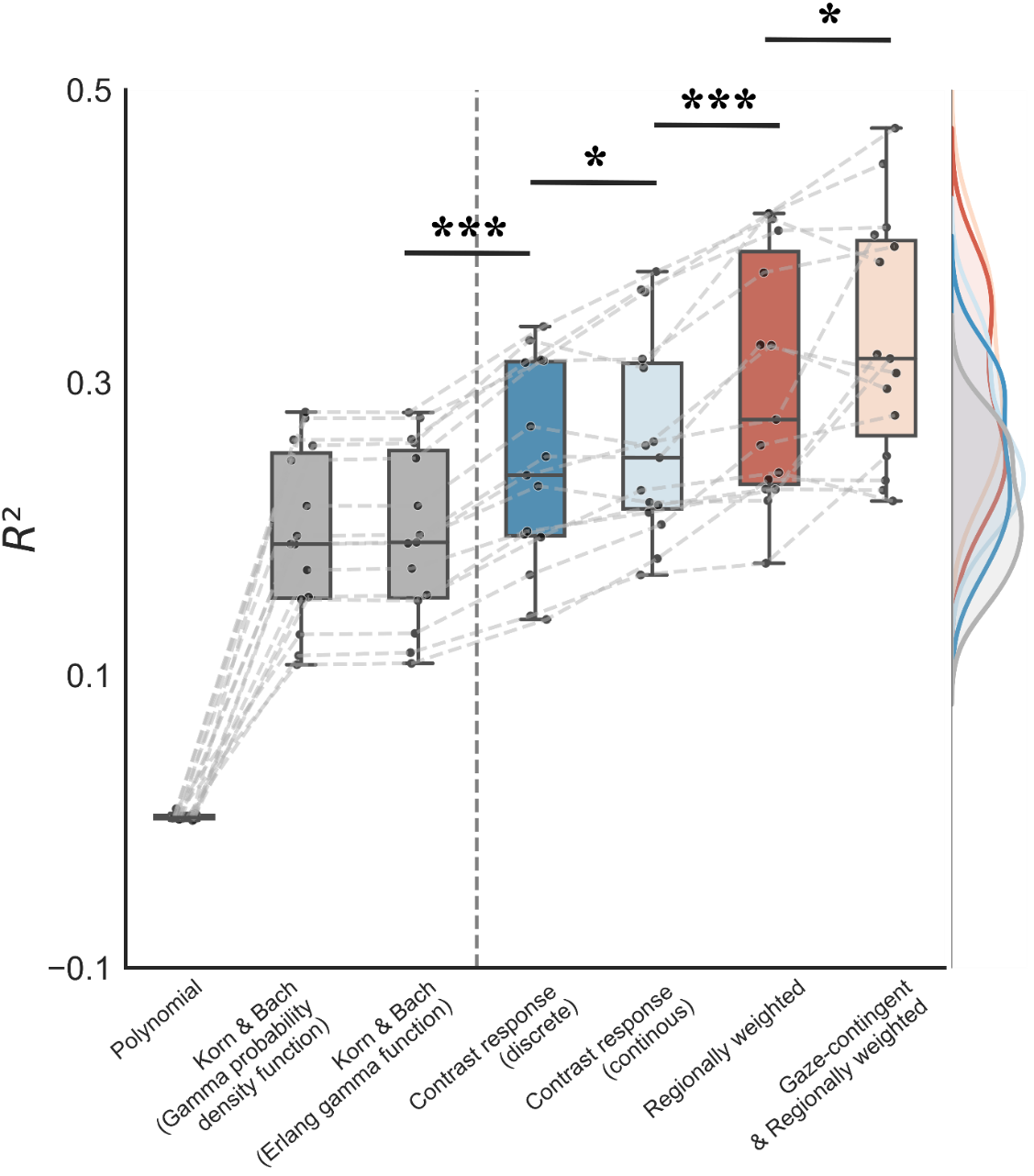

Supplement: Supplementary file 1 — Find the supplementary material via https://osf.io/qvn64/. (DOCX 1860 kb) [file 13428_2023_2292_MOESM1_ESM.docx]
